# Longitudinal assessment and clinical implications of treatment expectations in an outpatient pain centre: evaluation of the GEEE in patients with chronic pain

**DOI:** 10.1136/bmjopen-2024-097959

**Published:** 2026-05-03

**Authors:** Liv Henrich, Lukas A Basedow, Dustin Maser, Katharina Schmidt, Ulrike Bingel, Winfried Rief

**Affiliations:** 1 Department of Clinical Psychology and Psychotherapy, Philipps-Universität Marburg, Marburg, Germany; 2 Department of Neurology, Center for Translational Neuro- and Behavioral Sciences (C-TNBS), University Medicine Essen, University of Duisburg-Essen, Essen, Germany

**Keywords:** Chronic Pain, Pain management, Clinical Relevance, Clinical Decision-Making, Follow-Up Studies

## Abstract

**Abstract:**

**Objectives:**

There is evidence that treatment expectations predict treatment outcomes in pain management and other clinical conditions. However, translating these insights into clinical practice remains challenging: it is difficult to measure the multifaceted construct of expectations across diverse medical and psychological treatment modalities. Furthermore, little is known about how prior treatment experiences shape different expectation domains. A unified assessment approach is lacking, limiting comparability across studies and clinical contexts. The Generic Rating Scale for Previous Treatment Experiences, Treatment Expectations, and Treatment Effects (GEEE) seeks to overcome these limitations. The present study aims to explore the GEEE in a naturalistic clinical sample of people seeking treatment for chronic pain, which may provide preliminary evidence for its validity and applicability. An additional exploratory aim is to examine whether the GEEE is suitable for predicting treatment outcomes longitudinally in this clinical setting.

**Design:**

Prospective longitudinal observational study with three measurement time points (baseline, 3 weeks and 16 weeks).

**Setting:**

Specialised outpatient pain treatment centre in Germany.

**Participants:**

The baseline sample comprised 219 patients with chronic pain, follow-up data were available from 140 participants at 3 weeks and 108 participants at 16 weeks, constituting the longitudinal subsamples.

**Primary and secondary outcome measures:**

Primary outcomes were prior treatment experiences, current treatment expectations and treatment effects, which were assessed using the GEEE, as well as clinical outcomes of pain intensity and pain-related disability. Secondary measures included desire for pain relief, depression and anxiety symptoms, which were analysed in correlational tests to assess construct validity. Trajectories of treatment expectations and clinical outcomes were examined longitudinally, and it was assessed if baseline expectations predicted clinical outcomes over time.

**Results:**

Regarding validation, at baseline, improvement expectations correlated weakly with expectations of worsening and side effects (Rho≈−0.14 to −0.15, *p*=0.028–0.034). Negative previous treatment experiences were associated with current expectations of worsening and side effects (Rho≈0.35–0.41, *p*≤0.002). The GEEE items on current treatment effects correlated with changes in clinical outcomes (pain intensity and pain-related disability; |r|≈0.17–0.56, all *p*≤0.043). Regarding prediction, treatment expectations remained stable, while pain intensity (η²G=0.076, *p*<0.001) and pain-related disability (η²G=0.037, *p*<0.001) decreased over time. Regression models predicting subjective improvement were significant at 3 weeks (*R²*=0.18) and 16 weeks (*R²*=0.29), with baseline improvement expectations emerging as a significant predictor. Models predicting pain intensity and disability at 16 weeks were also significant (*R²*≈0.50–0.59), and higher baseline improvement expectations were independently associated with better outcomes.

**Conclusion:**

We found supporting evidence for the validity of the GEEE and its applicability in longitudinal clinical research. Improvement expectations as measured with the GEEE at baseline predicted better treatment outcomes, while previous negative treatment experiences correlated with current treatment expectations. The findings underscore the value of assessing and addressing expectations and prior treatment experiences to optimise treatment outcomes.

**STRENGTHS AND LIMITATIONS OF THIS STUDY**

- Data were collected longitudinally in a real-world clinical setting, enhancing ecological and external validity.
- The sample consisted of patients with chronic pain recruited from a specialised outpatient treatment centre, enhancing relevance for clinical practice.
- This sampling strategy may limit the generalisability of the findings to broader clinical populations.
- Substantial loss to follow-up and limited variance in some variables (Generic Rating Scale for Previous Treatment Experiences, Treatment Expectations, and Treatment Effects (GEEE) expectation of worsening, GEEE expectation of side effects and desire for pain relief) may have limited statistical power and generalisability.

## Introduction

 Expectation effects have long been regarded as a ‘nuisance’ in placebo-controlled clinical trials and have thus been neglected as predictors of treatment outcomes.^1^,^3^ Yet, countless studies have demonstrated that treatment expectations contribute to clinical outcomes in various conditions^4^,^8^ and are one of the main drivers of placebo and nocebo effects.^25 9^,^13^

The field of pain research represents an excellent example to illustrate that treatment expectations predict treatment outcomes across various pain stimuli and interventions.^5 8 14^ Expectations seem to not merely alter subjective experiences, but can also influence endogenous physiological processes—such as the release of endogenous opioids in the case of placebo hypoalgesia (ie, reduced pain perception induced by expectation, without an active pharmacological treatment).^11 15^ Interestingly, expectations not only modulate the effect of inert substances, but also of active treatments, for example, pharmacological interventions. For instance, positive expectations have even been shown to enhance the analgesic effect of opioids,^1116^,^18^ while negative expectations have been found to reduce or eliminate analgesic effects and increase the occurrence of side effects.^19 20^ Consequently, treatment expectations can be an important clinical target to improve treatment outcomes.^5^

Despite the enormous clinical potential of treatment expectations, translating research insights into practice is challenging. Expectations are a multifaceted construct and can be contingent on the specific medical condition, clinical context, treatment options and time frame. To date, there is no established unified assessment tool that can easily be used to measure treatment expectations across various treatment modalities.^21 22^

Due to its multidimensional nature, existing measurement tools vary greatly and encompass different aspects of the broader construct of ‘treatment expectations’. For instance, treatment expectations can be positive, that is, the hope for symptom improvement, or negative, that is, the fear of symptom worsening or side effects.^23 24^ However, the majority of assessment tools only measure positive expectations while neglecting negative expectations, even though the latter may independently modulate overall treatment outcomes.^12 22^

Additionally, little is known about the trajectory of treatment expectations or the potential impact of prior treatment experiences in routine care samples. Existing measurement tools are often either not sufficiently generic or too complex to assess various aspects of expectations, and thus not suitable for more frequent measurements in diverse clinical settings. To facilitate the systematic measurement of expectation effects, a versatile, time-efficient instrument is needed that integrates smoothly into clinical routines and allows for tracking expectations and outcomes over time. Using such a tool in clinical practice should foster our understanding of expectations as predictors of health outcomes, ensuring greater consistency and reliability in future research.

The Generic Rating Scale for Previous Treatment Experiences, Treatment Expectations, and Treatment Effects (GEEE)^23^ is a novel, generic assessment tool that can be applied before, during and after treatment and across various treatment settings. The scale captures expectations towards treatments, previous treatment experiences, as well as a subjective and generic evaluation of current treatment outcomes. In each of these domains, it assesses improvement, worsening and side effects through single items, making it both economical and practical for clinical use. In cross-sectional studies, the GEEE has shown promising results in terms of identifying dysfunctional treatment expectations.^25 26^

The aim of the present study is two-fold: (1) to validate the GEEE in a real-world clinical setting, and (2) to explore the role of treatment expectations over time, including their value in predicting clinical treatment outcomes. To address the first aim, we examined relationships among GEEE-measured expectations (improvement and worsening, side effects) and other pain-relevant measures to evaluate aspects of construct validity. To address the second, exploratory aim, we assessed associations between previous treatment experiences and pre-treatment expectations, explored changes in expectations and clinical outcomes during treatment, and tested whether baseline expectations predicted later clinical outcomes.

## Methods

### Design

In this longitudinal study using a consecutive clinical sample, self-reported data were collected as part of diagnostic screening and therapy monitoring at three time points (baseline, 3-week and 16-week follow-up).

### Participants

Participants were recruited from the Interdisciplinary Pain Center at the University Hospital Essen, Germany. All participants were informed about the purpose of standard therapy monitoring and provided informed consent prior to data collection.

### Ethical approval

The study was approved by the Ethics Committee of the Faculty of Medicine, University of Duisburg-Essen (reference: 21–10263 BO). To protect confidentiality, all data were anonymised before being shared with the researchers working on this study.

### Data management

Data were collected using the PainPool platform by smart-q (uhb Software GmbH, St. Wolfgang, Germany,https://www.smart-q.de/ed-portfolio/painpool/), which is commonly used in pain management settings, and stored on campus servers with internal data protection. On initial contact, participants received an email with a link to the PainPool interface including the informed consent form.

### Procedure

At each assessment point, participants received a link to the questionnaires. All assessments were completed on the devices of the participants. Data were collected from 10 September 2021 to 13 June 2022. The study analyses data from the three data collection points:

#### Baseline assessment

The initial assessment of pain treatment expectations (T1; baseline) happened several days (on average within 2 weeks) before the patient’s first scheduled appointment at the pain centre. During the first appointment, patients consulted with a physician specialised in pain management and received outpatient pain treatment, in most cases consisting of pain medication and physiotherapy prescriptions. If indicated, additional recommendations, such as psychological treatments or relaxation training, were provided. Additionally, patients were scheduled for follow-up consultations at 3 and 6 months to reassess and, if necessary, adjust their treatment.

#### Follow-up assessments

The follow-up assessments were conducted 3 weeks (T2) and 16 weeks (T3) after the baseline assessment.

### Materials

As part of the standard procedures at the pain centre, several pain-related instruments are collected for ongoing treatment monitoring. Only the instruments relevant to the present study are reported here.

#### Outcomes

##### Pain intensity

Pain intensity was assessed using the pain intensity items from the Graded Chronic Pain Scale (GCPS) developed by Von Korff *et al*.^27^ Items were rated on a scale from 0 (‘no pain’) to 10 (‘pain as bad as could be’) and averaged to create a composite score that encompassed maximum and average pain over the prior 4 weeks as well as current pain intensity. Pain intensity was assessed at all time points.

##### Pain-related disability

Pain-related disability was assessed using the German version of the Pain Disability Index (PDI),^28^ which evaluates current disability across seven daily life domains (eg, family obligations, hobbies and social life) on a scale from 0 (‘no disability’) to 10 (‘complete disability’). The total score can range from 0 to 70, with higher scores indicating greater disability. A broadened version of the PDI showed high internal consistency (Cronbach’s alpha=0.93) in a German sample.^29^ Pain-related disability was assessed at all time points.

##### Treatment expectations

The GEEE was used to assess three domains: previous treatment experiences, current treatment expectations and current treatment effects with regard to pain treatment at the pain centre.^23^ Each domain comprises three items (improvement, worsening and side effects), rated on an 11-point scale (eg, 0 = ‘no improvement’, 10 = ‘greatest improvement imaginable’; appendix D). Items were analysed individually, using average scores. The subscale on previous treatment experiences was divided into medication, physiotherapy and psychological treatment; only patients who had had previous experience with the respective treatment provided ratings for that modality. By contrast, the subscales on current expectations and current treatment effects referred to the pain treatment at the centre in general (ie, ‘How much improvement in your symptoms do you expect due to the pain treatment?’), independent of the specific modalities recommended or received. Expectation items were assessed at all time points, the previous experience subscale at baseline (T1), and the treatment effects subscale at the two follow-ups (T2 and T3).

##### Desire for pain relief

Desire for pain relief was measured using the German translation of the following visual analogue scale item from Banozic and Beljan^30^ : ‘how strong is your desire for pain relief?’ Responses range from 0 (‘no desire’) to 10 (‘most intense desire’). This item was assessed at all time points.

### Other predictors

#### Depression and anxiety

Depression and anxiety symptoms were measured using the German language short version of the Depression, Anxiety, and Stress Scale (DASS).^31 32^ This 21-item scale assesses symptoms on a 4-point Likert scale from 0 (‘did not apply to me at all’) to 3 (‘applied to me very much or most of the time’) referring to the past week, with a cut-off score of 10 for depression and 6 for anxiety.^33^ The DASS has shown high internal consistency, with Cronbach’s alphas of at least 0.91 for depression and 0.78–0.82 for anxiety.^31^ The DASS was assessed at baseline.

#### Incapacity to work

Incapacity to work among participants was operationalised using the disability days items from the GCPS by Von Korff *et al*.^27^ The score represents the number of days in the last 3 months in which a patient was not able to work, go to school or manage household activities because of pain. Incapacity to work was assessed at all time points.

### Statistical analysis

As this study used a consecutive clinical sample, no a priori sample size calculation was conducted. The available sample size was determined by the number of eligible patients during the study period, rather than by power considerations for the present analyses. Analyses are complete case analyses, based on the baseline sample for cross-sectional analyses and on the longitudinal subsamples for analyses involving follow-up data.

The data were processed using R Studio (Posit, PBC, Boston, MA, USA; version 2022.07.2)^34^ and the analyses were performed using Jamovi (The Jamovi project, Sydney, NSW, Australia; version 2.5.3.0).^34 35^

#### Validity

To evaluate the validity of the GEEE, we conducted correlation and regression analyses based on the baseline and longitudinal samples. Analyses of the baseline dataset were used to examine aspects of construct validity: intercorrelations among the GEEE expectation items assessed discriminant (improvement vs worsening) and convergent validity (worsening vs side effects), while correlations with depression tested discriminant validity. Analyses of the longitudinal subsets addressed item validity, through correlations between GEEE treatment effect items and changes in clinical outcomes (pain intensity and PDI), and criterion validity, specifically predictive validity, through regression analyses testing whether baseline expectations predicted outcomes at follow-up.

#### Treatment expectations over time: antecedents, trajectories and prediction of outcomes

To explore the longitudinal role of treatment expectations, we first examined the associations between baseline expectations and previous treatment experiences (medication, physical therapy and psychological treatments) using Spearman rank correlations. These analyses aimed to assess whether prior treatment experiences were related to the initial expectations of improvement, worsening or side effects in patients.

To evaluate changes in treatment expectations (improvement, worsening and side effects) and clinical outcomes (pain-related disability and pain intensity), we conducted repeated measures analyses of variance (ANOVAs) with one within-subjects factor (time: baseline, T2 and T3), followed by Tukey-corrected post-hoc comparisons.

To test whether baseline expectations, as measured with the GEEE, predicted clinical outcomes (pain-related disability; pain intensity; and GEEE: current improvement, worsening and side effects) at the two follow-ups, we performed multiple regression analyses for each outcome with Benjamini-Hochberg correction at the follow-ups. In each regression, 11 baseline predictors (age, gender, anxiety and depression, GEEE expectations of improvement, worsening and side effects, incapacity for work, desire for pain relief, and pain intensity and pain-related disability) were entered as independent variables in one step. Effect-size sensitivity analyses were conducted for the regression models at both follow-ups.^36^

## Results

### Demographic characteristics

At baseline, the sample (n=219, 59.5% female) had an average age of 53.4 years (SD=15.2; n=215). Of the patients, 85.84% reported back pain (lumbar, thoracic or cervical), and the rest experienced other pain types, including migraines. Overall, 75% of the sample had a chronic pain diagnosis (F45.41, pain persisting for more than 6 months) at baseline, while the remaining patients were referred to as chronic pain patients without official diagnosis. In terms of prior experience with different pain treatment modalities, 58.5% reported prior experience with medication, 66.8% with physical therapy and 25.3% with psychological treatments. For anxiety and depression symptoms, 33.6% scored above the DASS anxiety cut-off and 32.7% scored above the depression cut-off. At baseline, 219 patients completed the variables of interest, constituting the baseline sample. The longitudinal subsamples included 140 patients at the first follow-up and 108 at the second. Sample characteristics for all time points are reported in table 1. As shown in table 1, strong floor effects were observed for the expectation of worsening and expectation of side effects scales, while a pronounced ceiling effect was evident for desire for pain relief, indicating restricted variance for these measures.

**Table 1:** Sample characteristics

Measure	T1 (baseline) n=219 M (SD)	T2 (3 weeks) n=140 M (SD)	T3 (16 weeks) n=108 M (SD)
Anxiety (DASS subscale; 0–21)	4.42 (4.22) 3 (1-7)*	–	–
Depression (DASS subscale; 0–21)	7.60 (5.23)	–	–
Pain-related disability (PDI; 0–70)	37 (15.2)	35.90 (16.8)	28.20 (18.6)
Pain intensity (0–100)	70 (16.1)	60.9 (19.3)	52.90 (23.1)
Desire for pain relief (0–10)	9.38 (1.38) 10 (9–10)*	9.06 (1.71) 10 (9–10)*	8.30 (2.66) 10 (8–10)*
Incapacity for work (0–90)	37.4 (34.8)	36.3 (35.5)	22.5 (32.8) 5 (0–30)*
Expectation improvement (GEEE; 0–10)	7.42 (2.48)	7.12 (2.51)	7.19 (2.69)
Expectation worsening (GEEE; 0–10)	0.57 (1.58) 0 (0–0)*	0.91 (1.99) 0 (0–1)*	0.72 (1.71) 0 (0–0)*
Expectation side effects (GEEE; 0–10)	0.15 (2.19) 1 (0–3)*	2.12 (2.54) 1 (0–3)*	1.93 (2.13) 1 (0–3)*
Current improvement (GEEE; 0–10)	–	2.37 (2.61)	3.90 (2.93)
Current worsening (GEEE; 0–10)	–	1.24 (2.36) 0 (0–1)*	1.58 (2.63) 0 (0–2)*
Current side effects (GEEE; 0–10)	–	2.31 (2.72)	1.98 (2.33) 1.5 (0–3)*

* For skewed variables, both mean (SD) and median (25th–75th percentile) are reported

DASS, Depression, Anxiety, and Stress Scale; GEEE, Generic Rating Scale for Previous Treatment Experiences, Treatment Expectations, and Treatment Effects; M, mean; PDI, Pain Disability Index.

#### Dropout analyses

Dropout analyses indicated no baseline differences between completers and non-completers at T2. At T3, however, non-completers reported higher baseline pain intensity, depression, anxiety and desire for pain relief (all *p*<0.05; appendix A, table A1 and A2).

### Validity

#### GEEE expectations

At baseline, improvement expectations showed small negative correlations with expectations of worsening (Rho(217)=−0.15, *p*=0.028) and side effect expectations (Rho(217)=−0.14, *p*=0.034), which may tentatively support discriminant validity, although the restricted variance observed for these scales may have attenuated or artificially influenced correlation estimates. Additionally, expectations of worsening and of side effects were positively associated with one another (Rho(217)=0.31, *p*<0.001), which may support convergent validity under the same constraints.

None of the three expectation items correlated significantly with depression scores, indicating discriminant validity. Only side effect expectations correlated with anxiety (Rho(212)=0.16, *p*=0.02). All baseline expectations were unrelated to demographic characteristics.

#### GEEE current treatment effects and clinical outcome change

To evaluate the treatment effect items, we correlated their values with changes in clinical outcomes. At T2, the GEEE current improvement item correlated negatively with change in pain intensity (r(138)=−0.37, *p*<0.001) and change in pain-related disability (r(138)=−0.171, *p*=0.043), indicating that greater perceived improvement was associated with reductions in pain intensity and pain-related disability. Conversely, the GEEE current worsening item correlated positively with change in pain-related disability (r(138)=0.228, *p*=0.007) and the GEEE current side effects item also correlated positively with change in pain-related disability (r(138)=0.18, *p*=0.033), indicating that increases in perceived worsening and side effects were associated with greater increases in pain-related disability. A similar pattern emerged at T3: the current improvement item correlated negatively with change in pain intensity (r(104)=−0.564, *p*<0.001) and change in pain-related disability (r(104)=−0.512, *p*<0.001); the current worsening item correlated positively with change in pain intensity (r(104)=0.292, *p*=0.002) and change in pain-related disability (r(104)=0.293, *p*=0.002); and current side effects correlated positively with change in pain-related disability (r(104)=0.208, *p*=0.032). All of these results provide supporting evidence for the validity of the current treatment effect items of the GEEE.

### GEEE treatment expectations: antecedents, trajectories and prediction of outcomes

#### Correlations: GEEE expectations, prior experience and related variables

Prior experiences of pain worsening from medication, physical therapy or psychological treatments were associated with stronger expectations of worsening for the upcoming treatment (medication experience: Rho(125)=0.411, *p*<0.001; physiotherapy experience: r(143)=0.346, *p*<0.001; psychological treatment experience: Rho(53)=0.414, *p*=0.002; appendix B). The GEEE improvement expectation item correlated significantly with desire for pain relief at baseline (Rho(217)=0.345, *p*<0.001), at T2 (Rho(138)=0.331, *p*<0.001), and at T3 (r(106)=0.309, *p*=0.001).

#### Repeated measures ANOVAs: expectations and clinical outcomes over time

##### GEEE expectations

**Figure 1:**
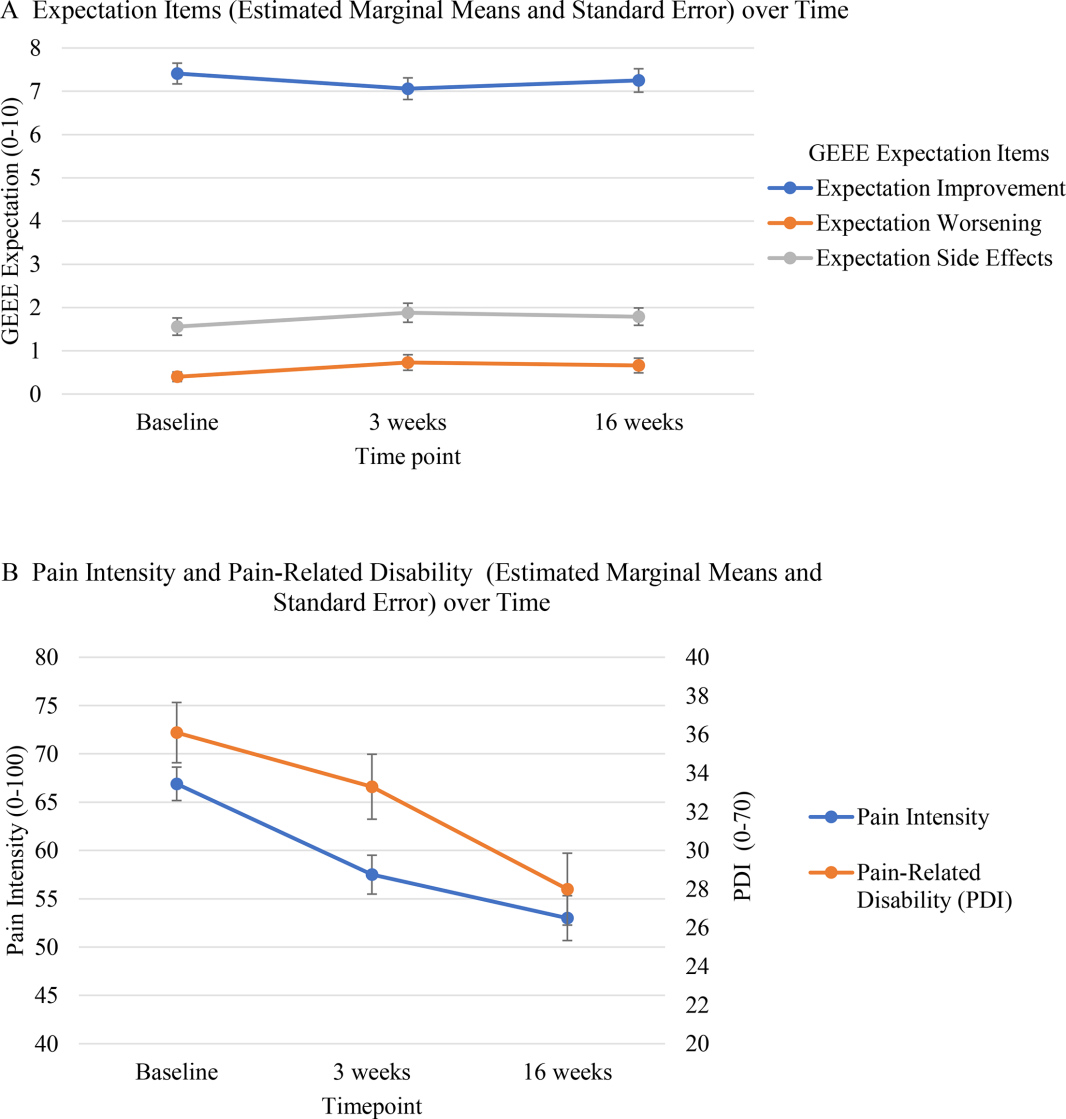
GEEE expectation items, pain intensity and pain-related disability over time. (A) Expectation items: estimated marginal means (±SE) at T1, T2 and T3. (B) Pain intensity and pain-related disability: estimated marginal means (±SE) at the same time points to facilitate comparison. GEEE, Generic Rating Scale for Previous Treatment Experiences, Treatment Expectations, and Treatment Effects.

Neither improvement expectations (*F*(2, 98)=0.794, *p*=0.454, η^2^G=0.003), expectations of worsening (χ^2^(2)= 4.14, *p*=0.126), nor side effect expectations (χ^2^(2)=4.14, *p*=0.126) showed significant differences across the time points (table 2; figure 1A).

**Table 2:** Estimated marginal means and standard errors of expectation items, pain intensity and pain-related disability over time

Measure	Baseline	3 weeks	16 weeks
	**M (SE**)	**M (SE**)	**M (SE**)
Expectation improvement (GEEE)	7.41 (0.24)	7.06 (0.25)	7.25 (0.27)
Expectation worsening (GEEE)	0.40 (0.11)	0.73 (0.18)	0.66 (0.17)
Expectation side effects (GEEE)	1.56 (0.20)	1.88 (0.22)	1.79 (0.20)
Pain intensity	66.9 (1.73)	57.5 (2.01)	53 (2.32)
Pain-related disability (PDI)	36.1 (1.56)	33.3 (1.68)	28 (1.86)

GEEE, Generic Rating Scale for Previous Treatment Experiences, Treatment Expectations, and Treatment Effects; M, estimated marginal mean; PDI, Pain Disability Index; T1, baseline; T2, after 3 weeks; T3, after 16 weeks.

##### Pain intensity

Pain intensity significantly decreased over the three time points (figure 1B), *F*(1.91, 98)=34.2, *p*<0.001, η^2^G=0.076, with post-hoc analyses indicating significant differences between the three time points (T1–T2: t=5.83; *p*<0.001; T1–T3: t=7.21; *p*<0.001; T2–T3: t=4.5; *p*<0.03).

##### Pain-related disability

Pain-related disability significantly decreased over the three time points (figure 1B; *F*(2, 98)=23.6, *p*<0.001, η^2^G=0.037, with post-hoc analyses confirming significant differences between T1 and T3 and between T2 and T3 (T1–T3: t=6.2; *p*<0.001; T2–T3: t=4.81; *p*<0.001).

##### Linear mixed model analyses

Linear mixed model analyses yielded the same pattern of results, supporting the findings from the complete case repeated-measures ANOVAs (appendix E).

### Regressions: baseline expectations predict clinical outcomes

Only significant models are reported. For all regression results, see Online supplemental appendix C. Restricted variance in some scales (GEEE expectation of worsening, GEEE expectation of side effects and desire for pain relief) should be considered when interpreting these analyses. For the T3 analyses, it should be noted that participants lost to follow-up had higher baseline pain, depression, anxiety and desire for pain relief, indicating potential attrition bias in these models. Effect-size sensitivity analyses (α=0.05, 80% power, 11 predictors) indicated sensitivity to moderate effects at the first follow-up (n=135; minimum detectable Cohen’s f^2^=0.14) and continued sensitivity to moderate effects at the second follow-up despite attrition (n=102; f^2^=0.18).

#### GEEE current treatment effects

The regression model for subjective improvement at T2 was significant, *F*(11, 123)=2.38, *p*=0.014, *R*^2^=0.176, adjusted *R*^2^=0.102, Cohen’s f^2^=0.214. Higher improvement expectations, side effect expectations and pain-related disability at baseline predicted greater perceived symptom improvement at T2. Conversely, higher incapacity for work scores at baseline predicted lower perceived improvement at T2 (table 3).

**Table 3:** Model coefficients GEEE subjective improvement at T2

Predictor at baseline	Estimate (B)	SE	T	p	Standardised estimate (ß)
(Intercept)*	5.3599	2.08452	2.571	0.011	
Age	−0.0252	0.01541	−1.632	0.105	−0.1425
Pain	−0.0256	0.01609	−1.589	0.115	−0.1634
Incapacity for work	**−0.0152**	**0.00706**	**−2.148**	**0.034**	**−0.2031**
Pain-related disability (PDI**)**	**0.0506**	**0.02120**	**2.385**	**0.019**	**0.2916**
Depression (DASS)	−0.0346	0.06668	−0.518	0.605	−0.0691
Anxiety (DASS)	0.0195	0.07604	0.257	0.798	0.0325
Expectation improvement (GEEE**)**	**0.1839**	**0.09175**	**2.005**	**0.047**	**0.1731**
Expectation worsening (GEEE)	−0.1694	0.17279	−0.980	0.329	−0.0873
Expectation side effects (GEEE**)**	**0.2383**	**0.10862**	**2.194**	**0.030**	**0.2029**
Desire for pain relief	−0.2625	0.20861	−1.259	0.211	−0.1216
Gender					
male—female	−0.7275	0.46224	−1.574	0.118	−0.2759

Significant predictors in bold.

* Represents reference level

B, coefficient estimate; DASS, Depression, Anxiety, and Stress Scale; GEEE, Generic Rating Scale for Previous Treatment Experiences, Treatment Expectations, and Treatment Effects; PDI, Pain Disability Index; T1, baseline; T2, after 3 weeks; T3, after 16 weeks.

The regression model for subjective improvement at T3 was also significant, *F*(11, 90)=3.41, *p*=0.01, *R*^2^=0.294, adjusted *R*^2^=0.208, Cohen’s f^2^=0.416. Higher improvement expectations at baseline predicted greater perceived symptom improvement at T3, higher expectations of worsening at baseline predicted greater perceived improvement at T3, and higher anxiety at baseline predicted lower perceived improvement at T3 (table 4).

**Table 4:** Model coefficients GEEE subjective improvement at T3

Predictor at baseline	Estimate (B)	SE	T	P	Standardised estimate (ß)
(Intercept*)	7.08348	2.38505	2.970	0.004	
Age	−0.02411	0.01832	−1.316	0.191	−0.1241
Pain	−0.01574	0.02018	−0.780	0.438	−0.0889
Incapacity for work	−0.00120	0.00849	−0.141	0.888	−0.0142
Pain-related disability (PDI)	−0.01651	0.02680	−0.616	0.539	−0.0850
Depression (DASS)	0.05235	0.09060	0.578	0.565	0.0902
Anxiety (DASS)	**−0.29921**	**0.11330**	**−2.641**	**0.010**	**−0.3629**
Expectation improvement (GEEE)	**0.32798**	**0.12370**	**2.651**	**0.009**	**0.2518**
Expectation worsening (GEEE)	**0.76773**	**0.28307**	**2.712**	**0.008**	**0.2547**
Expectation side effects (GEEE)	0.21723	0.14346	1.514	0.133	0.1401
Desire for pain relief	−0.28119	0.24127	−1.165	0.247	−0.1245
Gender					
male—female	−0.05828	0.57610	−0.101	0.920	−0.0196

Significant predictors in bold.

* Represents reference level

B, coefficient estimate; DASS, Depression, Anxiety, and Stress Scale; GEEE, Generic Rating Scale for Previous Treatment Experiences, Treatment Expectations, and Treatment Effects; PDI, Pain Disability Index; T1, baseline; T2, after 3 weeks; T3, after 16 weeks.

#### Prediction of pain intensity at T2

The regression model was significant, *F*(11, 123)=11.1, *p*=0.01, *R*^2^=0.498, adjusted *R*^2^=0.453, indicating a large effect size, Cohen’s f^2^=0.992. Higher pain intensity at baseline was the only significant predictor of higher pain intensity at T2. All model coefficients are provided in online suplemental appendix C, table 5.

#### Prediction of pain-related disability at T2

The regression model was significant, *F*(11, 123)=16.0, *p*=0.01, *R*^2^=0.589, adjusted *R*^2^=0.552, indicating a large effect size, Cohen’s f^2^=1.433. Higher pain-related disability at baseline predicted higher pain-related disability at T2 and was the only significant predictor. All model coefficients are provided in online supplemental appendix C, table 6.

#### Prediction of pain intensity at T3

The regression model was significant, *F*(11, 92)=8.24, *p*=0.01, *R*^2^=0.496, adjusted *R*^2^=0.436, indicating a large effect size, Cohen’s f^2^=0.984. Higher anxiety and pain-related disability at baseline predicted higher pain intensity at T3. Higher improvement expectations at baseline predicted lower pain intensity at T3. The strongest predictor of pain intensity at T3 was pain intensity at baseline. All model coefficients are provided in online supplemental appendix C, table 7.

#### Prediction of pain-related disability at T3

The regression model was significant, *F*(11, 92)=12.1, *p*=0.01, *R*^2^=0.591, adjusted *R*^2^=0.543, indicating a large effect size, Cohen’s f²=1.444. Higher anxiety at baseline predicted higher pain-related disability at T3. Higher improvement expectations predicted lower pain-related disability at T3. The strongest predictor of pain-related disability at T3 was pain-related disability at baseline. All model coefficients are provided in online supplemental appendix C, table 8.

## Discussion

In this study, we investigated the validity and clinical usefulness of the novel generic expectation assessment scale (GEEE) in chronic pain patients at a pain care centre. We found evidence supporting the validity of the GEEE. We also observed that current expectations of improvement, worsening and side effects were only weakly associated, indicating the multidimensional structure of treatment expectations. Surprisingly, previous treatment success was not associated with baseline improvement expectations, whereas experience of previous worsening and side effects was associated with baseline expectations of worsening and side effects. Notably, higher baseline improvement expectations predicted greater subjective improvement and lower pain intensity as well as pain-related disability at follow-up, underlining the clinical usefulness of assessing treatment expectations.

### Validity

Our study provides further support for the validity of the GEEE as an instrument for measuring treatment expectations over time, in line with previous research that linked the GEEE to established measures such as the Credibility/Expectancy Questionnaire and the Treatment Expectation Questionnaire.^25 26^ At baseline, inter-item correlations provided preliminary internal evidence of discriminant (improvement vs worsening/side effects) and convergent validity (worsening vs side effects), while the absence of associations with depression further support discriminant validity.

Significant correlations between GEEE treatment effect items and changes in clinical outcomes confirm the convergent validity of the items; greater perceived improvement was associated with reduced pain intensity and pain-related disability, while subjective worsening corresponded to poorer clinical outcomes. In addition, regression analyses showed that the GEEE current improvement item shared a similar pattern of significant predictors—most notably improvement expectations—with pain intensity and pain-related disability outcomes, supporting the criterion-related (predictive) validity of the instrument.

#### Baseline treatment expectations

Interestingly, our data showed only a weak negative correlation between expectations of improvement and expectations of worsening, supporting the notion that these are two distinct dimensions rather than two ends of the same scale. This aligns with previous mixed findings, with one study reporting a weak negative correlation in patients with endometriosis^26^ and others finding no significant relationship in patients with chronic pain^25^ and in diverse clinical samples.^37^ Overall, these findings highlight the importance of assessing positive and negative expectations separately.

Our finding that at baseline, higher improvement expectations were associated with lower side effect expectations may seem intuitive. However, research using the GEEE, as well as other studies, reported either no correlation or an inverse relationship, with higher improvement expectations being associated with higher side effect expectations, potentially reflecting a ‘no pain, no gain’ mindset.^26 38 39^ We found positive correlations between expectations of worsening and expectations of side effects, suggesting that patients associate the anticipation of worsening symptoms with side effects, a finding that is consistent with existing literature.^2638^,^41^ Additionally, higher anxiety was associated with stronger side effect expectations, also consistent with previous findings on medical interventions.^42^

### GEEE treatment expectations: antecedents, trajectories and prediction of outcomes

#### Previous experience and treatment expectations

The previous experience scale of GEEE revealed that past experiences of worsening were associated with higher expectations of worsening and of side effects, while past experiences of improvement were not related to current improvement expectations. Another recent study using the GEEE also reported that past worsening and past side effects predicted current side effect expectations.^40^ Our results further align with research on carry-over effects, which suggests that negative experiences have a more substantial impact on expectation formation than positive experiences and are more prone to generalisation compared with positive treatment experiences (‘better safe than sorry effect’).^43^,^45^

#### Trajectories of treatment expectations and clinical outcomes

In terms of the trajectory of expectations, we found that treatment expectations remained relatively stable over the course of the study, despite a reduction in pain intensity and pain-related disability in the same period. This finding corresponds to previous studies that reported stable expectations during pain treatments.^46^,^48^ However, experience with treatment should also shape treatment expectations^40 45^ and the correlations we found between previous negative treatment experiences and baseline expectations partially support this notion. One potential explanation could be that past treatments may influence current expectations differently than ongoing treatments, or that a change in treatment setting may affect expectations. The lack of identified changes may also be due to ceiling effects for positive expectations, as most patients had optimistic expectations, which were often reinforced by treatment success.

#### Treatment expectations predict clinical outcomes

Finally, the need to thoroughly assess baseline expectations is supported by our findings regarding the prediction of treatment outcomes. Notably, improvement expectations consistently predicted symptom reduction across the different outcomes at the 16-week follow-up. However, improvement expectations did not predict outcomes at the 3-week follow-up. One possible explanation is that interventions need more time to show strong effects (eg, treatment with antidepressants, physical therapy and psychological treatments). Furthermore, verbal suggestions about when treatment effects would occur may have shaped the temporal expectations of patients, such that improvements were anticipated later rather than earlier.^49^,^51^ Interestingly, expectations of worsening also predicted greater subjective improvement at the 16-week follow-up, perhaps reflecting a subgroup of positively surprised patients.^45^ One mechanism might be that expecting positive outcomes indeed improves treatment outcomes, akin to a placebo response,^5^ while another mechanism may reflect a ‘better safe than sorry’ strategy, insofar as patients maintain expectations of worsening to prepare themselves for negative outcomes, thereby minimising potential disappointment.^45^ Patients can have hopes and concerns about treatments at the same time, and both may affect treatment outcomes. Given the modest explanatory power of the model, floor effects for expectations of worsening and selective attrition at T3, the findings should be interpreted with caution. Although the results of the regression analyses need to be replicated using the GEEE in similar samples before firm conclusions can be drawn, our findings are supported by a large and consistent body of literature indicating that expectation effects are important determinants of treatment responses in diverse contexts.^4 5 38 45 52 53^

### Strengths and limitations

First, our study extends previous validation work by including longitudinal aspects in the assessment of the validity of GEEE in a clinical pain management context. However, as this investigation was based on a naturalistic clinical sample rather than a study designed exclusively for validation purposes, the evidence for validity must be interpreted within this context. A dedicated validation study, with more controlled conditions, could further substantiate these findings.

Second, the data collection was integrated into standard medical procedures at the pain centre, enhancing the external validity of the findings. As such, the study provides a link between experimental placebo studies on placebo mechanisms in pain and the clinical application of expectation effects. A limitation of this study is that the sample consisted of patients with and without formal chronic pain diagnosis. While this may limit generalisability, it reflects real-world clinical conditions in tertiary pain care and thus enhances external validity. Future studies could distinguish between chronic and acute pain patients to clarify potential differences in expectation effects. In this sample, low expectations of worsening and side effects suggest strong floor effects, while desire for pain relief showed a ceiling effect, indicating restricted variance that may distort associations in correlational and regression analyses and limit the robustness of findings involving these scales. Future research could explore alternative measurement strategies or subgroup analyses to better capture variability in expectations of worsening and side effects and to identify patient characteristics associated with these expectations. Further studies in diverse samples are needed to examine whether outcomes meet or diverge from the initial expectations of patients and to evaluate the potential effect of expectation violation vs congruency for predicting treatment outcomes.

Third, to better understand expectations and placebo/nocebo responses for different treatment modalities, future research should adopt a more nuanced approach, incorporating additional measurement time points and separate expectation assessments for various treatment types (eg, pharmacological, psychological and physical therapy), as the treatment type might interact with patient preferences and decision-making. The timing of previous treatment experiences may also be an important factor to consider.

Fourth, although expectations are increasingly recognised as dynamic^44^ and our repeated measurements represent a step toward understanding changes over time, our results indicated relative stability across the three time points. Smaller within-person fluctuations may nevertheless have occurred but were not detectable with our design. Future studies could apply ecological momentary assessment to examine such fine-grained dynamics.

Finally, attrition was substantial, which is common in naturalistic longitudinal data collection; at T3, non-completers had higher baseline symptom severity in terms of pain intensity, depression and anxiety and desire for pain relief, indicating that this was a more seriously affected subgroup. This pattern suggests potential attrition bias, such that the T3 analyses may over-represent patients with better baseline functioning. The results may therefore overestimate the strength of associations between predictors and outcomes that are more pronounced in patients with better baseline functioning and may not generalise to more severely affected patients. Moreover, because the regression analyses at T3 were based on a reduced sample with multiple predictors, the risk of model overfitting cannot be ruled out. Sensitivity analyses indicated adequate power to detect moderate effects at both follow-ups, while smaller effects may have gone undetected. Given the relatively small sample size, these predictive analyses should be interpreted as exploratory and hypothesis-generating rather than hypothesis-driven, as no prespecified hypotheses were tested. Our findings on the association between expectations and treatment outcomes are correlational rather than causal in nature, as we did not manipulate expectations directly. To draw causal conclusions about this association, experimental studies are required.

## Conclusion

The present study demonstrates the potential of the GEEE as a valid assessment tool that can easily be applied in routine clinical practice and longitudinal research. Importantly, the GEEE assesses previous experiences and their valence, as well as current expectations regarding improvement, worsening and side effects—which have been identified as valuable and partially independent components that can predict clinical outcomes. Our findings highlight carry-over effects of negative experiences, emphasising the importance of capturing and considering them in treatment planning. The stability of treatment expectations throughout the study further suggests that a potentially sensitive time to modulate expectations is before treatment begins—an approach that has shown promising results in other studies.^45 54^ Crucially, the results confirm that pre-treatment expectations of improvement, as measured with the GEEE, significantly predict subsequent subjective improvement and clinical outcomes related to pain and disability. Overall, these findings highlight the feasibility and clinical relevance of assessing baseline expectations and tracking their development over time. This could inform personalised treatment decisions and enhance treatment outcomes in the future. An adaptation of the GEEE in other clinical conditions and treatment settings is thus encouraged.

## Supplementary material

10.1136/bmjopen-2024-097959 online supplemental file 1

10.1136/bmjopen-2024-097959 online supplemental file 2

10.1136/bmjopen-2024-097959 online supplemental file 3

10.1136/bmjopen-2024-097959 online supplemental file 4

10.1136/bmjopen-2024-097959 online supplemental file 5

## Data Availability

The datasets generated and/or analysed in the current study are available upon reasonable request.
