# Supplementary material for: Longitudinal assessment and clinical implications of treatment expectations in an outpatient pain centre: evaluation of the GEEE in patients with chronic pain
Source: BMJ Open. 2026 May 3;16(4):e097959. doi: 10.1136/bmjopen-2024-097959 (PMC13141194; doi:10.1136/bmjopen-2024-097959)
Supplement: online supplemental file 1 [file bmjopen-16-4-s001.docx]

**APPENDIX A**.

Additional Information about Statistical Analyses

**Statistical Analysis**

***Skewed Variables***

Based on skewness values exceeding ±1, the following variables were considered skewed and are reported with both mean (SD) and median (25th–75th percentile):

- **Baseline:**
  - Desire for pain relief
  - Anxiety
  - Expectation of worsening
  - Expectation of side effects
- **T2:**
  - Desire for pain relief
  - Expectation of worsening
  - Expectation of side effects
  - Current worsening
- **T3:**
  - Desire for pain relief
  - Incapacity to work
  - Expectation of worsening
  - Expectation of side effects
  - Current worsening
  - Current side effects

***Dropout Analysis Tables***

**Table A1.**

*Baseline Differences between Completers and Non-completers at T2*

| **Variable** | **Completers M (SD)** | **Non-completers M (SD)** | **p** |
| --- | --- | --- | --- |
| Age | 54.24 (14.88) | 51.79 (15.82) | .265 |
| Pain intensity | 69.19 (16.84) | 71.44 (14.60) | .303 |
| Work incapacity | 36.41 (35.45) | 39.13 (33.83) | .575 |
| PDI | 37.53 (15.16) | 35.98 (15.42) | .472 |
| Depression | 7.17 (5.27) | 8.33 (5.10) | .115 |
| Anxiety | 4.28 (4.39) | 4.65 (3.94) | .533 |
| Expectancy – improvement | 7.43 (2.51) | 7.41 (2.44) | .946 |
| Expectancy – worsening | 0.48 (1.39) | 0.73 (1.87) | .290 |
| Expectancy – side effects | 1.69 (2.25) | 1.90 (2.10) | .498 |
| Desire for pain relief | 9.33 (1.40) | 9.47 (1.36) | .470 |

Note. Values are means with standard deviations in parentheses. Group differences were tested using Welch’s t-tests.

**Table A2.**

*Baseline Differences between Completers and Non-completers at T3*

| **Variable** | **Completers M (SD)** | **Non-completers M (SD)** | **p** |
| --- | --- | --- | --- |
| Age | 54.14 (15.20) | 52.59 (15.32) | .458 |
| Pain intensity | 66.79 (16.66) | 73.12 (14.90) | .003 |
| Work incapacity | 35.63 (35.71) | 39.10 (34.00) | .463 |
| PDI | 35.94 (15.28) | 37.97 (15.20) | .324 |
| Depression | 6.40 (5.09) | 8.73 (5.13) | .001 |
| Anxiety | 3.47 (3.57) | 5.31 (4.60) | .001 |
| Expectancy – improvement | 7.43 (2.32) | 7.41 (2.63) | .973 |
| Expectancy – worsening | 0.42 (1.06) | 0.72 (1.95) | .152 |
| Expectancy – side effects | 1.61 (1.94) | 1.92 (2.42) | .299 |
| Desire for pain relief | 9.19 (1.52) | 9.57 (1.22) | .041 |

Note. Values are means with standard deviations in parentheses. Group differences were tested using Welch’s t-tests.

***Correlation Analyses***

Due to non-normal distribution of baseline variables (Shapiro-Wilk: p < .05), Spearman’s rank correlation was used for these variables.

***Repeated Measures ANOVAs***

Expectations of worsening were analyzed using Friedman's test due to the non-normal distribution. For the analysis of pain intensity, the assumption of sphericity was violated and a Huynh-Feldt correction was applied.

***Regression Analysis***

**Assumptions.** Assumptions for the regression analyses were examined. Autocorrelation was tested using the Durbin–Watson statistic, and multicollinearity was assessed via variance inflation factors (VIFs). Visual inspection of Q–Q plots and residual plots indicated no major deviations from normality or homoscedasticity.

Inspection of the data revealed that at baseline, desire for pain relief were skewed toward the higher end of the scale, whereas anxiety, expectation of worsening, and side-effect expectation were skewed toward the lower end. Log-transformations did not substantially improve the distributional properties; therefore, the direction of skewness should be considered when interpreting the results.

**Correction for Multiple Comparisons.** Given that several regression analyses were conducted across related outcomes, we applied a false discovery rate (FDR) correction on the main analyses using the Benjamini–Hochberg procedure to control for multiple comparisons.
