# Supplementary material for: Longitudinal assessment and clinical implications of treatment expectations in an outpatient pain centre: evaluation of the GEEE in patients with chronic pain
Source: BMJ Open. 2026 May 3;16(4):e097959. doi: 10.1136/bmjopen-2024-097959 (PMC13141194; doi:10.1136/bmjopen-2024-097959)
Supplement: online supplemental file 2 [file bmjopen-16-4-s002.docx]

**APPENDIX B.**

Correlation Matrices Prior Experience with Pain Treatment

**Table B1.**

*Correlation Matrix Prior Experience with Medication and Baseline Expectations (GEEE)*

| Measure | 1 | 2 | 3 | 4 | 5 | 6 |
| --- | --- | --- | --- | --- | --- | --- |
| 1. Prior improvement | - |  |  |  |  |  |
| 2. Prior worsening | 0.120  (127) | - |  |  |  |  |
| 3. Prior side effects | 0.133  (127) | 0.327***  (127) | - |  |  |  |
| 4. Expectation improvement | -0.085  (127) | -0.146  (127) | -0.115  (127) | - |  |  |
| 5. Expectation worsening | 0.101  (127) | 0.411***  (127) | 0.260**  (127) | -0.148*  (219) | - |  |
| 6. Expectation side effects | 0.062  (127) | 0.280**  (127) | 0.267**  (127) | -0.144*  (219) | 0.307***  (219) | - |

Note. * p < .05, ** p < .01, *** p < .001, Spearmans Rho (*N*), GEEE = Generic Rating Scale for Previous Treatment Experiences, Treatment Expectations, and Treatment Effects.

**Table B2.**

*Correlation Matrix Prior Experience with Physical Therapy and Baseline Expectations (GEEE)*

| Measure | 1 | 2 | 3 | 4 | 5 | 6 |
| --- | --- | --- | --- | --- | --- | --- |
| 1. Prior improvement | - |  |  |  |  |  |
| 2. Prior worsening | 0.306***  (145) | - |  |  |  |  |
| 3. Prior side effects | 0.194*  (145) | 0.639***  (145) | - |  |  |  |
| 4. Expectation improvement | -0.127  (145) | -0.170*  (145) | -0.014  (145) | - |  |  |
| 5. Expectation worsening | 0.172*  (145) | 0.346***  (145) | 0.252**  (145) | -0.148*  (219) | - |  |
| 6. Expectation side effects | 0.216**  (145) | 0.209*  (145) | 0.217**  (145) | -0.144*  (219) | 0.307***  (219) | - |

Note. * p < .05, ** p < .01, *** p < .001, Spearmans Rho (*N*), GEEE = Generic Rating Scale for Previous Treatment Experiences, Treatment Expectations, and Treatment Effects.

**Table B3.**

*Correlation Matrix Prior Experience with Psychological Treatment and Baseline Expectations (GEEE)*

| Measure | 1 | 2 | 3 | 4 | 5 | 6 |
| --- | --- | --- | --- | --- | --- | --- |
| 1. Prior improvement | - |  |  |  |  |  |
| 2. Prior worsening | -0.303*  (55) | - |  |  |  |  |
| 3. Prior side effects | 0.235  (55) | 0.448***  (55) | - |  |  |  |
| 4. Expectation improvement | 0.084  (55) | -0.017  (55) | -0.361**  (55) | - |  |  |
| 5. Expectation worsening | 0.183  (55) | 0.414**  (55) | 0.258  (55) | -0.148*  (219) | - |  |
| 6. Expectation side effects | 0.047*  (55) | 0.394**  (55) | 0.320*  (55) | -0.144*  (219) | 0.307***  (219) | - |

Note. * p < .05, ** p < .01, *** p < .001, Spearmans Rho (*N*), GEEE = Generic Rating Scale for Previous Treatment Experiences, Treatment Expectations, and Treatment Effects.
