# Supplementary material for: Longitudinal assessment and clinical implications of treatment expectations in an outpatient pain centre: evaluation of the GEEE in patients with chronic pain
Source: BMJ Open. 2026 May 3;16(4):e097959. doi: 10.1136/bmjopen-2024-097959 (PMC13141194; doi:10.1136/bmjopen-2024-097959)
Supplement: online supplemental file 3 [file bmjopen-16-4-s003.pdf]

APPENDIX C.

Additional Regression Analyses

GEEE Current Treatment Effects

Predicting GEEE Worsening at T2. The regression model was not significant, $F(11, 123) = 1.20$, $p = .37$, $R^2 = .097$, adjusted $R^2 = .016$, indicating a small effect size, Cohen's $f^2 = .107$. Experienced worsening at T2 was not significantly predicted by the included variables.

Predicting GEEE Side Effects at T2. The regression model was not significant, $F(11, 123) = 0.72$, $p = 0.72$, $R^2 = .060$, adjusted $R^2 = -.023$, Cohen's $f^2 = .063$. The experience of side effects at T2 was not significantly predicted by the included variables.

Predicting GEEE Worsening at T3. The regression model was significant, $F(11, 90) = 2.57$, $p = .012$, $R^2 = .239$, adjusted $R^2 = .146$, Cohen's $f^2 = .314$. None of the predictors individually were significant.

Predicting GEEE Side Effects at T3. The regression model was not significant, $F(11, 90) = 1.03$, $p = .47$, $R^2 = .112$, adjusted $R^2 = .004$, Cohen's $f^2 = .126$. The experience of side effects at T3 was not significantly predicted by the included variables.

Regression: Pain Intensity and Pain-Related Disability (PDI) at T2 (after 3 Weeks)

Table 5.

Model Coefficients - Regression with Outcome 'Pain Intensity' at T2

Predictor at Baseline	Estimate (B)	SE	t	p	Std. Estimate (ß)
(Intercept [a])	-2.55130	11.8783	-0.2148	0.830	
Age	0.10233	0.0878	1.1651	0.246	0.07941
Depressive symptoms (DASS)	0.16046	0.3800	0.4223	0.674	0.04395
Anxiety (DASS)	0.34845	0.4333	0.8041	0.423	0.07946
Expectation improvement (GEEEE)	-0.71139	0.5228	-1.3606	0.176	-0.09172
Expectation worsening (GEEE)	0.33882	0.9846	0.3441	0.731	0.02391
Expectation side effects (GEEE)	-0.35802	0.6189	-0.5785	0.564	-0.04177
Desire for pain relief	1.59184	1.1887	1.3391	0.183	0.10100
Incapacity for work	0.00277	0.0402	0.0688	0.945	0.00508
Pain-related disability (PDI)	0.12871	0.1208	1.0655	0.289	0.10166
**Pain intensity**	**0.60136**	**0.0917**	**6.5589**	**< .001**	**0.52651**
Gender					
male – female	-1.54428	2.6340	-0.5863	0.559	-0.08021

Model Coefficients - Regression with Outcome 'Pain Intensity' at T2

Predictor at Baseline	Estimate (B)	SE	t	p	Std. Estimate (ß)

Note. [a] Represents reference level, B = coefficient estimate, ß = standardized estimate, GEEE = Generic Rating Scale for Previous Treatment Experiences, Treatment Expectations, and Treatment Effects, PDI = Pain Disability Index, T1 = Baseline, T2 = after 4 weeks, T3 = after 3 months, significant results in bold.

Table 6.

Model Coefficients - Regression with Outcome 'Pain-Related Disability' at T2

Predictor at Baseline	Estimate (B)	SE	t	p	Std. Estimate (ß)
(Intercept [a])	-13.8078	9.3525	-1.476	0.142	
Age	0.0733	0.0692	1.060	0.291	0.0653
Depressive symptoms (DASS)	-0.2214	0.2992	-0.740	0.461	-0.0697
Anxiety symptoms (DASS)	0.6708	0.3412	1.966	0.052	0.1758
Expectation improvement (GEEEE)	-0.2095	0.4117	-0.509	0.612	-0.0310
Expectation worsening (GEEE)	0.5562	0.7752	0.717	0.474	0.0451
Expectation side effects (GEEE)	-0.7395	0.4873	-1.517	0.132	-0.0991
Desire for pain relief	1.4810	0.9359	1.582	0.116	0.1080
Incapacity for work	0.0621	0.0317	1.961	0.052	0.1310
**Pain-related disability (PDI)**	**0.5811**	**0.0951**	**6.110**	**< .001**	**0.5273**
Pain intensity	0.1284	0.0722	1.778	0.078	0.1291
Gender					
male – female	0.2234	2.0739	0.108	0.914	0.0133

Note. [a] Represents reference level, B = coefficient estimate, ß = standardized estimate, GEEE = Generic Rating Scale for Previous Treatment Experiences, Treatment Expectations, and Treatment Effects, PDI = Pain Disability Index, T1 = Baseline, T2 = after 4 weeks, T3 = after 3 months, significant results in bold.

Regressions: Pain Intensity and Pain-Related Disability (PDI) at T3 (after 16 Weeks)

Table 7.

Model Coefficients - Regression with Outcome 'Pain Intensity' at T3

Predictor at Baseline	Estimate (B)	SE	t	p	Std. Estimate (ß)
(Intercept [a])	-8.0022	15.7047	-0.510	0.612	
Age	0.1969	0.1206	1.633	0.106	0.1290
Depressive symptoms (DASS)	-0.4339	0.5864	-0.740	0.461	-0.0951
**Anxiety symptoms (DASS)**	**1.5065**	**0.7449**	**2.022**	**0.046**	**0.2319**
**Expectation improvement (GEEE)**	**-2.5769**	**0.8097**	**-3.182**	**0.002**	**-0.2515**
Expectation worsening (GEEE)	-1.2369	1.8632	-0.664	0.508	-0.0521
Expectation side effects (GEEE)	-0.9333	0.9374	-0.996	0.322	-0.0768
Desire of pain relief	2.8345	1.5744	1.800	0.075	0.1597

Model Coefficients - Regression with Outcome 'Pain Intensity' at T3

Predictor at Baseline	Estimate (B)	SE	t	p	Std. Estimate (ß)
Incapacity for work	-0.0317	0.0551	-0.575	0.566	-0.0484
**Pain-related disability (PDI)**	**0.4565**	**0.1739**	**2.625**	**0.010**	**0.2999**
**Pain intensity**	**0.4182**	**0.1303**	**3.210**	**0.002**	**0.3005**
Gender					
male – female	-1.6503	3.7429	-0.441	0.660	-0.0711

Note. [a] Represents reference level, B = coefficient estimate, ß = standardized estimate, GEEE = Generic Rating Scale for Previous Treatment Experiences, Treatment Expectations, and Treatment Effects, PDI = Pain Disability Index, T1 = Baseline, T2 = after 4 weeks, T3 = after 3 months, significant results in bold.

Table 8.

Model Coefficients - Regression with Outcome 'Pain-Related Disability' at T3

Predictor at Baseline	Estimate (B)	SE	t	p	Std. Estimate (ß)
(Intercept [a])	-8.9996	11.1877	-0.804	0.423	
Age	0.1506	0.0859	1.753	0.083	0.12469
Depressive symptoms (DASS)	-0.2767	0.4178	-0.662	0.509	-0.07669
**Anxiety symptoms (DASS)**	**1.1429**	**0.5307**	**2.154**	**0.034**	**0.22241**
**Expectation improvement (GEEEE)**	**-1.8211**	**0.5768**	**-3.157**	**0.002**	**-0.22466**
Expectation worsening (GEEE)	-0.9090	1.3273	-0.685	0.495	-0.04841
Expectation side effects (GEEE)	-0.0731	0.6678	-0.110	0.913	-0.00761
Desire of pain relief	1.2088	1.1216	1.078	0.284	0.08610
Incapacity for work	0.0371	0.0393	0.945	0.347	0.07149
**Pain-related disability (PDI)**	**0.6785**	**0.1239**	**5.477**	**< .001**	**0.56337**
Pain intensity	0.0528	0.0928	0.569	0.570	0.04800
Gender					
male – female	0.9027	2.6663	0.339	0.736	0.04919

Note. [a] Represents reference level, B = coefficient estimate, ß = standardized estimate, GEEE = Generic Rating Scale for Previous Treatment Experiences, Treatment Expectations, and Treatment Effects, PDI = Pain Disability Index, T1 = Baseline, T2 = after 4 weeks, T3 = after 3 months, significant results in bold.
