# Supplementary material for: Longitudinal assessment and clinical implications of treatment expectations in an outpatient pain centre: evaluation of the GEEE in patients with chronic pain
Source: BMJ Open. 2026 May 3;16(4):e097959. doi: 10.1136/bmjopen-2024-097959 (PMC13141194; doi:10.1136/bmjopen-2024-097959)
Supplement: online supplemental file 4 [file bmjopen-16-4-s004.docx]

**Appendix D.**

**Generic rating scale for previous treatment experiences, treatment expectations, and treatment effects (G-EEE)**

**Expectations**

The following section is about what **expectations** you have regarding the treatment:

1. **How much improvement in** **your symptoms** **do you expect due to the pain treatment?**

0 = No improvement - 10 = Greatest improvement imaginable

2. **How much worsening of** **your symptoms** **do you expect due to the pain treatment?**

0 = No worsening – 10 = Greatest worsening imaginable

3. **How much discomfort / how many side effects do you expect due to the pain treatment?**

0 = No discomfort – 10 = Greatest discomfort imaginable

**Previous experiences**

4. The following section is about what **previous treatment experiences** you have had with (pain medication/ physiotherapy/ psychological treatment):

**I have never experienced this treatment** *(continue with question 8)*.

**I have experienced this treatment (almost) daily in the last 12 months.**

**I have experienced this treatment on more than 10 days in the last 12 months.**

**I have experienced this treatment on approx. 5-10 days in the last 12 months.**

**I have experienced this treatment on approx. 1-4 days in the last 12 months.**

**I have not experienced this treatment in the last 12 months, but I have experienced it before.**

5. **How much improvement in** **your symptoms** **have you experienced due to the treatment** with pain medication/ physiotherapy/ psychological treatment **in the past?**

0 = No improvement – 10 = Greatest improvement imaginable

6. **How much worsening of** **your symptoms** **have you experienced due to the treatment** with pain medication/ physiotherapy/ psychological treatment **in the past?**

0 = No worsening – 10 = Greatest worsening imaginable

7. **How much discomfort / how many side effects have you experienced due to the treatment** with pain medication/ physiotherapy/ psychological treatment **in the past?**

0 = No discomfort – 10 = Greatest discomfort imaginable

**Current effects**

The following section is about what **changes you have experienced since participating in this study:**

8. **How much improvement in** **your symptoms** **have you experienced since participating?**

0 = No improvement – 10 = Greatest improvement imaginable

9. **How much worsening of** **your symptoms** **have you experienced since participating?**

0 = No worsening – 10 = Greatest worsening imaginable

10. **How much discomfort / how many side effects have you experienced since participating?**

0 = No discomfort – 10 = Greatest discomfort imaginable
