# Supplementary material for: Longitudinal assessment and clinical implications of treatment expectations in an outpatient pain centre: evaluation of the GEEE in patients with chronic pain
Source: BMJ Open. 2026 May 3;16(4):e097959. doi: 10.1136/bmjopen-2024-097959 (PMC13141194; doi:10.1136/bmjopen-2024-097959)
Supplement: online supplemental file 5 [file bmjopen-16-4-s005.docx]

# Appendix E.

# Linear Mixed-Effects Model (LMM) Analyses

To address reviewer feedback and to account for missing data resulting from participant dropout, linear mixed-effects models (LMMs) were conducted for each dependent variable across the three measurement time points. LMMs allow for the inclusion of all available observations without requiring complete data from all participants, thereby providing a more robust estimation of longitudinal changes. Results for each variable are presented below. These results should be interpreted in light of the assumption that missing data are missing at random (MAR), which may be violated given that dropout was associated with baseline symptom severity.

## Improvement Expectations

A linear mixed-effects model was conducted to examine changes in improvement expectations across the three measurement time points. The fixed effect of time was not significant, F(2, 246) = 1.11, *p* = .332, indicating that participants’ improvement expectations remained stable over time. Parameter estimates showed no significant differences between time points (T2 − T1: b = −0.31, SE = 0.22, t = −0.93, *p* = .16; T3 − T1: b = −0.22, SE = 0.24, t = −0.93, *p* = .35). Post-hoc pairwise comparisons with Bonferroni correction confirmed that none of the contrasts reached significance (T1–T2: *p* = .34; T1–T3: *p* = .62; T2–T3: *p* = .93). Overall, the model suggests that improvement expectations did not change meaningfully across the three time points.

## Expectations of Worsening

A linear mixed-effects model was conducted to examine changes in expectations of worsening across the three measurement time points. The fixed effect of time approached significance, F(2, 246) = 2.45, *p* = .088. Parameter estimates suggested that expectations of worsening were slightly higher at T2 compared to T1 (b = 0.37, SE = 0.17, t = 2.16, *p* = .032), whereas the difference between T3 and T1 was not significant (b = 0.26, SE = 0.17, t = 1.53, *p* = .13). Post-hoc pairwise comparisons with Bonferroni correction indicated that none of the contrasts reached significance (T1–T2: *p* = .08; T1–T3: *p* = .28; T2–T3: *p* = .79). Overall, these findings suggest a small, short-term increase in expectations of worsening from baseline to the second time point, which was not sustained at the final assessment.

## Side-Effect Expectations

A linear mixed-effects model was conducted to examine changes in side-effect expectations across the three measurement time points. The fixed effect of time was not significant, F(2, 246) = 2.32, *p* = .101, indicating that participants’ side-effect expectations remained largely stable over time. Parameter estimates suggested a small increase from T1 to T2 (b = 0.43, SE = 0.20, t = 2.15, *p* = .032), while the difference between T3 and T1 was not significant (b = 0.30, SE = 0.24, t = 1.26, *p* = .21). Post-hoc pairwise comparisons confirmed that none of the contrasts reached significance after Bonferroni correction (T1–T2: *p* = .08; T1–T3: *p* = .42; T2–T3: *p* = .78). Overall, the model indicates that side-effect expectations showed a slight but non-significant increase at the second measurement, returning to baseline levels at follow-up.

## Pain Intensity

A linear mixed-effects model was conducted to examine changes in pain intensity across the three measurement time points. The fixed effect of time was significant, F(2, 246) = 44.9, *p* < .001, indicating a marked decrease in pain intensity over time. Parameter estimates showed that pain intensity significantly decreased from T1 to T2 (b = −8.67, SE = 1.29, t = −6.75, *p* < .001) and from T1 to T3 (b = −14.42, SE = 1.57, t = −9.18, *p* < .001). Post-hoc pairwise comparisons confirmed that pain intensity differed significantly between all three time points (T1–T2: *p* < .001; T1–T3: *p* < .001; T2–T3: *p* < .001). These findings indicate a steady and statistically significant reduction in pain intensity from baseline to the two follow-ups.

## Pain-Related Disability (PDI)

A linear mixed-effects model was conducted to examine changes in pain-related disability across the three measurement time points. The fixed effect of time was significant, F(2, 246) = 23.3, *p* < .001, indicating that pain-related disability decreased significantly over time. Parameter estimates showed that PDI scores decreased from T1 to T2 (b = −1.38, SE = 1.00, t = −1.38, *p* = .17) and from T1 to T3 (b = −7.26, SE = 1.13, t = −6.41, *p* < .001). Post-hoc pairwise comparisons confirmed that PDI scores at T3 were significantly lower than at both T1 and T2 (T1–T3: *p* < .001; T2–T3: *p* < .001), whereas the difference between T1 and T2 was not significant (*p* = .35). Overall, these findings indicate a statistically significant reduction in pain-related disability between baseline and the final assessment.
