# Supplementary material for: Longitudinal assessment and clinical implications of treatment expectations in an outpatient pain centre: evaluation of the GEEE in patients with chronic pain
Source: BMJ Open. 2026 May 3;16(4):e097959. doi: 10.1136/bmjopen-2024-097959 (PMC13141194; doi:10.1136/bmjopen-2024-097959)
Supplement: Supplementary file 6 [file bmjopen-2024-097959.reviewer_comments.pdf]

PEER REVIEW HISTORY

BMJ Open publishes all reviews undertaken for accepted manuscripts. Reviewers are asked to complete a checklist review form and are provided with free text boxes to elaborate on their assessment. These free text comments are reproduced below.

ARTICLE DETAILS

Title (Provisional)

Longitudinal assessment and clinical implications of treatment expectations in an outpatient pain center: evaluation of the GEEE in patients with chronic pain

Authors

Henrich, Liv; Basedow, Lukas A.; Maser, Dustin; Schmidt, Katharina; Bingel, Ulrike; Rief, Winfried

---

VERSION 1 - REVIEW

**Reviewer**	**1**
**Name**	**Schemer , Lea**
**Affiliation**	**Rheinland-Pfälzische Technische Universität Kaiserslautern-Landau**
**Date**	**17-Apr-2025**
**COI**	**None**

The purpose of this study is to test the use of the Generic Rating Scale for Previous Treatment Experiences, Treatment Expectations, and Treatment Effects (GEEE) in outpatients at a specialized pain treatment center and to determine whether the GEEE is predictive of treatment outcome. Although the topic is highly relevant, I have the following methodological concerns and ideas for improvement.

1) Unfortunately, the way the paper is currently written gives the impression that the authors looked for correlations in a routine dataset and then interpreted their results post hoc. To give an example, in the introduction the authors state that their aim was to explore relationships between GEEE and other pain-related measures. In the results and discussion sections, the authors interpret the non-significant correlation between the GEEE baseline items as an indicator of discriminant validity, while they interpret the partially significant correlations with desire for pain relief as an indicator of convergent validity. Greater clarity at the outset about hypotheses and their rationale would greatly enhance the transparency and interpretability of the work.

2) I was also unsure about the primary intent of the authors. If their primary intent is to validate the GEEE, the baseline data could be used to assess convergent and divergent construct validity, while predictive analyses might be more appropriately framed in terms of criterion validity. This would position the study more clearly as a questionnaire validation. Alternatively, if the main objective is to assess whether expectations predict therapy

outcomes in routine care, the correlational analyses could be presented more as a methodological quality check. I personally find the part about testing temporal stability unconvincing, since temporal stability would have had to be tested, for example, by administering the same questionnaire two weeks apart. However, the distance between the measurements was quite large and the participants did receive treatment between surveys.

3) It is commendable that the authors collected longitudinal data in a routine care setting. However, the presentation of the sample size is somewhat misleading. While the authors report a total sample of 219 participants, only 140 and 108 individuals were available at the first and second follow-up, respectively. For the sake of transparency, I would prefer to speak of two sample subsets, e.g., a baseline sample (e.g., to examine discriminant and convergent construct validity) and the longitudinal sample set (e.g., to examine criterion validity).

4) The presentation of the GEEE was confusing to me. I have also looked at the citations provided, but I still have some unanswered questions. As I understand it, the core items of the GEEE are the ones about improvement, worsening, and side effects expectations. But if I want to use this questionnaire in research or practice, what do I do with these items? Do I score them individually? Or in combination with past or current experience? Am I looking at change scores?

In this study, things seem to have been even more complicated because different treatments were rated separately. Did all patients rate all treatments? Or only the ones that were recommended to them? Were the expectation items then averaged or looked at again separately? Including a clearer description, possibly supported by a visual overview or table of the assessed items, would be very helpful.

5) In my opinion, it would be better to limit the statistical analyses depending on the focus of the paper. It is also concerning that the authors apply a variety of statistical tests without giving any thought to correcting for alpha errors.

6) The discussion highlights that improvement and worsening/side-effect expectations only moderately correlate, suggesting they should be assessed separately. However, only improvement expectations significantly predicted pain intensity and disability at follow-up, which somewhat weakens their point. I have also open questions about why it is important to measure worsening and side effect expectations separately. Moreover, I am curious whether it would not be more relevant to look at individual changes, i.e., whether a person's pain improves or worsens more than expected. I think there is still a lot of potential in the data.

7) The limitations section refers to the dynamic nature of treatment expectations, which contradicts the statement of the paper that expectations were seemingly stable in this sample. This apparent contradiction is not addressed and could confuse readers. It may be advisable to either reconcile these points or consider omitting the stability analysis altogether, as mentioned earlier.

**Reviewer**	**2**
**Name**	**Camerone, Eleonora Maria**
**Affiliation**	**University of Oxford**

**Date**	**14-Aug-2025**
**COI**	**None**

Longitudinal assessment and clinical implications of treatment expectations in routine clinical settings: evaluation of the GEEE in patients with chronic pain.

This is an exciting and well-conducted study that seeks to bridge the gap between evidence on the powerful effects of treatment expectations from clinical trials and experimental research and their translation into real-world clinical practice. To this end, the authors explore the use of a new tool to measure treatment expectations (GEEE) in a pain management setting among patients seeking pain treatment (i.e., examining various correlations and stability over time), while also implementing a longitudinal design to test whether baseline treatment expectations predict later clinical outcomes. The study design is generally well-conceived, and the writing is clear and concise throughout. However, I have a few major and some minor comments that I recommend addressing to increase the rigour of the study.

Abstract.

1. *Line 11 (page 3)*. Specify the type of outpatients (e.g., pain sufferers or patients seeking pain treatment). I would not say chronic pain patients, because this is not the case for the whole sample.
2. *Line S (page 2). "The present study examines the use of this new instrument…"* - The term *examines* is rather unspecific. If the main aim of the study is to validate the GEEE scale (which remains unclear throughout the manuscript but becomes clearer in the Discussion, where validation appears to be a central aim), I would recommend stating this explicitly from the outset and using the term *validate* rather than *explore*.

Introduction.

3. *Line 10 (page 4)*. The term 'placebo analgesia' appears here for the first time; I suggest adding a brief definition and considering the more accurate term 'placebo hypoalgesia,' as it usually refers to reduced - not eliminated - pain.

Statistical Analyses.

4. How was missing data managed? Please specify

5. Sample size calculation. I could not find a description of the sample size calculation. Although the sample size is relatively large (n = 219), one of the primary aims is to predict clinical outcomes from baseline treatment expectations. Prediction models often require large samples to ensure adequate statistical power, and adequacy depends on the number of predictors. My understanding is that there are at least eight (more likely 10) predictors: age, gender, baseline DASS, GEEE expectations (potentially three predictors given the subscales), incapacity for work, desire for pain relief, and baseline pain intensity

and pain-related disability. I suggest making the number of predictors explicit. If no a priori sample size calculation was performed, please consider adding a post hoc power analysis or explaining how the sample size was determined.

6. Correlation Analyses. *Lines 27-28 (page 7).* The authors mention testing the correlation between the GEEE improvement expectation item and 'desire for pain relief' to assess convergent validity. However, I am not convinced by this approach, as I do not think the two measures are assessing the same construct. While these variables may or may not be correlated (and it is great that the authors test this), wanting pain relief (desire for pain relief) is not the same as expecting pain relief. In fact, previous experiences might influence these differently: for example, one might have very low expectations of pain relief while still having a strong desire to experience less pain. Please let me know if I am missing something here.

7. Correlation Analyses. *Line 28-2S (page 7).* The authors write: "*we assessed correlations (v) between GEEE current treatment effects and changes in clinical outcomes*". Could the authors clarify (1) why these correlations were run (e.g., as part of the GEEE validation strategy?) and (2) the exact time points involved? My understanding is that these are associations at each follow-up (T2, T3) between current perceived improvement, worsening, and side effects and changes from baseline (correct?). I suggest stating this explicitly in the Methods and framing them as validation analyses (if that is the case) to distinguish them from the predictive regression models.

8. Has the study been pre-registered?

Results.

9. Demographic Characteristics. *Line S (page 8).* Please specify how *chronic pain* was defined (e.g., pain persisting for longer than six months). Additionally, as only 74.89% of patients had chronic pain, could the authors clarify how the remaining participants were classified (e.g., acute pain)? I suggest adding details on eligibility criteria for participants in the methods section under the 'Participant' subsection (page 5).

10. Demographic Characteristics. *Lines 10-11 (page 8).* It is great that the authors reported prior experience with the pain treatment modalities of focus for this study (i.e., medication, physical treatment, and psychological treatment). However, it would also be informative to know whether patients had tried other types of treatments. Have the authors assessed this? If so, it would be helpful to include data such as the total number of pain treatments tried by patients.

11. Correlation Analyses - GEEE Expectation *(page 8)*. The rationale for conducting correlation analyses between GEEE expectation items at baseline is unclear. In my view, these analyses could represent a reasonable approach to assessing item validity. Could the authors clarify whether this was indeed their rationale? If so, I would recommend stating this explicitly in the text, as was done for the correlation between baseline expectation items and depression scores. Specifically, testing the correlation between improvement expectations and expectations of worsening appears to be a reasonable way to assess discriminant validity (please feel free to disagree). Similarly, the correlation between expectations of worsening and expectations of side effects seems a reasonable way to assess convergent validity, as both reflect negative treatment outcome expectations. Clarifying these points would, in my view, strengthen the manuscript and further advance the validation of the GEEE.

12. Repeated Measures ANOVAs – Pain Intensity and Pain-Related Disability. *Line 55 (page S) and line 5 (page 10)*. Were the post-hoc analyses corrected for multiple comparisons? Please specify.

Discussion – Overall, it's concise, well-structured, and truly exciting.

13. *Line 14 (page 12)*. The statement *"in a sample of patients with chronic pain"* seems inaccurate, as only 74.89% of participants suffer from chronic pain. Given the established role of expectations in chronic pain (e.g., inflexible expectations), the inclusion of a hybrid sample with both chronic and more acute (?) pain patients is an important limitation. I recommend clarifying in the Discussion (and in the limitations section) that the sample is mixed, explaining why non-chronic patients were included, and considering how this may affect interpretation. One possible - though sample-size-limiting - approach would be to re-run analyses excluding non-chronic patients.

14. *Lines 2C-27 (page 12)*. Not sure I agree with the following statement: "*The GEEE's improvement expectation item showed significant correlations with desire for pain relief across all time points, indicating convergent validity*". See comment 6 regarding correlation analyses and convergent validity (corr. GEEE improvement expectation item and 'desire for pain relief).

15. As indicated in the 'Validity' section of the Discussion *(page 12)*, one of the study's aims is to, at least in part, validate the GEEE instrument. I suggest making this explicit from the outset, as it is currently only partially clear.

16. When presenting the prediction results (page 13-14), the authors report only the significant findings at the 16-week follow-up, without discussing why the results differ at the 3-week follow-up (where only pain intensity/pain-related disability emerged as a significant predictor). It would be valuable to add a

brief comment on the potential role of temporal expectations and to explore why the 3- and 6- week outcomes differ. One possible explanation is that patients may not expect to improve shortly after treatment (e.g., at 3 weeks), but may anticipate treatment effects to manifest later (e.g., at 16 weeks). The following studies on temporal expectations may be relevant:

- Camerone, E. M., Piedimonte, A., Testa, M., Wiech, K., Vase, L., Zamfira, D. A., ... C Carlino, E. (2021). The effect of temporal information on placebo analgesia and nocebo hyperalgesia. *Psychosomatic Medicine*, *83*(1), 43-50.
- Camerone, E. M., Wiech, K., Benedetti, F., Carlino, E., Job, M., Scafoglieri, A., C Testa, M. (2021). 'External timing'of placebo analgesia in an experimental model of sustained pain. *European Journal of Pain*, *25*(6), 1303-1315.

**Reviewer**	**3**
**Name**	**Purtill, Helen**
**Affiliation**	**University of Limerick, Mathematics and Statistics**
**Date**	**21-Sep-2025**
**COI**	**None**

This study presents an interesting research topic in the evaluation and use of GEEE in patients with chronic pain.

1. Missing data (lost to follow-up)

The study design is longitudinal across 3 timepoints (baseline, T2 (>3 weeks), T3 (>16 weeks) but suffers from a high dropout (n = 219 at baseline, n = 140 at T2, n = 108 at T3). It would be of importance to examine whether those who dropped out of the study differed across the measured baseline characteristics (demographic, pain intensity, disability etc) to those who remained in the study. As over 50% of participants were no longer in the study at T3 this really needs to be acknowledge in the limitations, with careful consideration about how this may have affected the results. The Strengths and Limitations section pg 2,3 currently makes no acknowledgement of the potential effects of missingness and lost to follow-up. For example, it could be that those who are more motivated and more expressive are more likely to remain in the study until T3.

2. Presentation of Descriptive Statistics:

Table 1 should also include characteristics described at all timepoints (where they are measured) with sample sizes given in the column headings. At present there are no descriptives given for current treatment experiences at T2 and T3 or GEEE at T2 and T3. In Table 1 means and SDs are given to summarise the numeric data but where the data is skewed (for example Expectation worsening and Expectation side effects look to be positively skewed) it should be summarised as median (IQR) or median (25th percentile,

75th percentile). Expectation of side effects and expectation of worsening appears to be very low at baseline so there would be floor effects - it would not be possible for someone to have a value lower than 0.

3. Correlation Analyses:

Correlations are presented which examine the strength of the baseline linear associations between GEEE scales, GEEE and previous treatment (but no consideration of when the previous treatment occurred), GEEE and depression. It may also be interesting to examine if any demographic characteristics (age, sex, back pain), baseline pain intensity, disability are associated with baseline GEEE scales - i.e. are there any participant characteristics associated with higher expectations?

Pg 9. Line 23 - 24: "All of these results confirm the validity of the current

treatment effect items of the GEEE.." - While correlation analysis may be consistent with or provide evidence for validity it can not alone cannot confirm the validity of a scale. Assessing the validity of a scale is a much broader task beyond the design and analyses in this study.

4. Repeated Measures ANOVA of GEEE, pain intensity and disability

Repeated measures ANOVA provides a complete case analysis and the results of this analysis are only relevant to those who have completed all 3 timepoints (less than half of the 219 who started). A linear mixed model analysis would provide an analysis of all participants who provided at least one measurement and should be used instead of repeated measures ANOVA or the limitation of using complete case analysis should be clearly stated.

5. Regression analyses: Assumptions of regression rely on the normality and constant variance of the residuals - this should be examined and reported on for the regression analyses.

Regression analysis of GEEE Current Treatment effects - the R-square for these models are very low reflecting low accuracy in any predictions for these outcomes.

Prediction of pain intensity and disability at times T2 and T3 provide results that are somewhat conflicting. At T2 only baseline pain predicts pain at T2, similarly for disability (only baseline disability predicts disability). But at T3 (when the sample size has reduced), Treatment expectation predicts pain, similarly for disability.

The sample size for these regression models at T3 is only 100 but there are 11 predictor variables, there is a risk of overfitting a regression model to a small sample.

The conclusions from these models need to be framed within an acknowledgement of a relatively small sample size with many predictor variables.

Other comments:

Pg 2: Line 15: "We utilized the GEEE as an economic tool to assess treatment expectations.." Needs to be rephrased as it reads like GEEE provides an economic assessment.

Pg 2, Line 17: "Improvement expectations correlated with desire for pain relief, indicating convergent validity." - but convergent validity is where two scales are designed to measure the same concept - but surely expectation of improvement and a desire for pain relief are different concepts? Also, the correlation shows only that they are weakly correlated (rho ≅ 3.5).

Pg 8, Line 22 - 23: "....Improvement expectations showed modest negative correlations with expectations of....", the rho values show small/weak correlations of -0.15 and -0.14 - these would not be considered modest.

VERSION 1 - AUTHOR RESPONSE

Reviewer: 1
Dr. Lea Schemer , Rheinland-Pfälzische Technische Universität Kaiserslautern-Landau
Comments to the Author:
The purpose of this study is to test the use of the Generic Rating Scale for Previous Treatment Experiences, Treatment Expectations, and Treatment Effects (GEEE) in outpatients at a specialized pain treatment center and to determine whether the GEEE is predictive of treatment outcome. Although the topic is highly relevant, I have the following methodological concerns and ideas for improvement.

1) Unfortunately, the way the paper is currently written gives the impression that the authors looked for correlations in a routine dataset and then interpreted their results post hoc. To give an example, in the introduction the authors state that their aim was to explore relationships between GEEE and other pain-related measures. In the results and discussion sections, the authors interpret the non-significant correlation between the GEEE baseline items as an indicator of discriminant validity, while they interpret the partially significant correlations with desire for pain relief as an indicator of convergent validity. Greater clarity at the outset about hypotheses and their rationale would greatly enhance the transparency and interpretability of the work.

- We thank the reviewer for this comment. We would like to emphasize that all analyses were conceptually grounded in the study's predefined aims. The study was designed with two aims in mind: (1) to validate the GEEE by examining its indicators of validity, mainly in the baseline sample and (2) to apply the instrument in a real-world clinical setting to explore its longitudinal associations with clinical outcomes. To address this concern, we have revised the abstract, introduction and the manuscript more broadly to make the study aims and associated analyses more explicit. We believe these clarifications now prevent any impression of post hoc interpretation and strengthen the transparency and rationale of our work.
- We acknowledge that the correlation between improvement expectations and desire for pain relief represent a relatively weak indicator of convergent validity and may be an indicator of construct validity more broadly, as the two constructs are distinct, yet theoretically related.
- We have reworded several sections of the manuscript to include the validation aspect more explicitly, clarify the two overarching aims of the study, and specify which analyses were conducted in relation to each aim.
- In line with the reviewer's third comment, we have adopted the terminology distinguishing between the baseline and longitudinal samples. The baseline sample primarily served the validation aim, whereas the longitudinal subsamples served the exploratory aim regarding the role of expectations over time and the assessment of predictive validity.
    - Abstract p. 2 lines 10-12: *"The present study aims to test the validity and applicability of this new instrument in patients seeking pain treatment. An additional exploratory aim is to assess the role of treatment expectations*

over time and to examine whether the GEEE is suitable for predicting treatment outcome longitudinally in this clinical setting."

- Introduction p.5 lines 7-14: "*The aim of the present study is two-fold: (1) to validate the GEEE in a real-world clinical setting, and (2) explore the role of treatment expectations over time, including their value in predicting clinical treatment outcomes. To address the first aim, we examined relationships among GEEE-measured expectations (improvement, worsening, side effects) and other pain-relevant measures to evaluate aspects of construct validity. To address the second, exploratory aim, we assessed associations between previous treatment experiences and pre-treatment expectations, explored changes in expectations and clinical outcomes during treatment, and tested whether baseline expectations predicted later clinical outcomes."*
- Methods, Statistical analysis on p. 7 and 8: *"**Validity** To evaluate the validity of the GEEE, we conducted correlation and regression analyses based on the baseline and longitudinal samples. Analyses of the baseline dataset were used to examine aspects of construct validity: intercorrelations among the GEEE expectation items assessed discriminant (improvement vs. worsening) and convergent validity (worsening vs. side effects), while correlations with depression tested discriminant validity. Associations between improvement expectations and desire for pain relief, analysed at all three time points, served as further indicators of construct validity, as these two theoretically related constructs were expected to show a positive association.. Analyses of the longitudinal subsets (T2 and T3) addressed item validity, through correlations between GEEE treatment effect items and changes in clinical outcomes (pain intensity, PDI), and criterion validity, specifically predictive validity, through regression analyses testing whether baseline expectations predicted outcomes at follow-up.*
  ***Treatment Expectations over Time** To explore the longitudinal role of treatment expectations, we first examined the associations between baseline expectations and previous treatment experiences (medication, physical therapy, and psychological treatments) using Spearman rank correlations. These analyses aimed to assess whether prior treatment experiences were related to patients' initial expectations of improvement, worsening, or side effects. To evaluate changes in treatment expectations (improvement, worsening, and side effects) and clinical outcomes (pain-related disability, pain intensity), we conducted repeated measures ANOVAs with one within-subjects factor (time: baseline, T2, and T3), followed by Tukey-corrected post-hoc comparisons. To test whether baseline expectations, as measured with the GEEE predict clinical outcomes (pain-related disability; pain intensity; GEEE: current improvement, worsening, and side effects) at the two follow-ups, we performed multiple regression analyses for each outcome with Benjamini-Hochberg correction at each follow-up. In each regression 11 predictors (age, gender, baseline anxiety and depression, GEEE expectations, incapacity for work, desire for pain relief, and baseline pain intensity and pain-related disability) were entered as independent variables in one step. Post-hoc power analyses were conducted for all regression models (36). (See Appendix A for additional information.)"*
- Results section on p.9 and p.10 is now divided into results relating to the validity aim (p.9) and the exploratory aim (treatment expectations over time on p. 10)
- Discussion p 13, lines 9-11: *" In this study, we investigated the validity and clinical utility of the novel generic assessment of expectation scale GEEE in chronic pain patients of a routine pain care center. We found evidence supporting the validity of the GEEE."*
- Discussion p. 13, lines 22-27: "*At baseline, inter-item correlations provided preliminary internal evidence of discriminant (improvement vs. worsening/side-effect) and convergent validity (worsening vs. side-effect),*

while the absence of associations with depression further supported discriminant validity. The GEEE's improvement expectation item consistently correlated with desire for pain relief, providing supportive—though not definitive—evidence of construct validity, acknowledging that the two constructs are theoretically related but not identical."

2) I was also unsure about the primary intent of the authors. If their primary intent is to validate the GEEE, the baseline data could be used to assess convergent and divergent construct validity, while predictive analyses might be more appropriately framed in terms of criterion validity. This would position the study more clearly as a questionnaire validation. Alternatively, if the main objective is to assess whether expectations predict therapy outcomes in routine care, the correlational analyses could be presented more as a methodological quality check. I personally find the part about testing temporal stability unconvincing, since temporal stability would have had to be tested, for example, by administering the same questionnaire two weeks apart. However, the distance between the measurements was quite large and the participants did receive treatment between surveys.

- We thank the reviewer for this thoughtful comment. We would like to clarify that the study was designed with a dual aim in mind: (1) to validate the GEEE, focusing on its psychometric properties such as convergent and discriminant validity, and (2) to explore the role of treatment expectations in a longitudinal, real-world clinical setting, including their value in predicting clinical outcomes. Because of the setting, the data collection points were embedded in the existing routine data collection points at the pain center, rather than by validation considerations of the study. The analyses of treatment expectations over time, which we called temporal stability analyses, mainly fall within this second, exploratory aim and are discussed in this context (p. 14, lines 24-34). We did not want to give the impression that the results should be interpreted as an indicator of test-retest reliability.
- To address the reviewer's concern, we have revised the manuscript to make the dual aim explicit consistently throughout the paper. We have also adopted the terminology suggested in comment 3 regarding baseline and longitudinal subsamples and clarified which sample was used to asses each aim. See responses to comment 1.
- In addition, when discussing the rmANOVA results we no longer call these analyses 'temporal stability analyses', as not to give the impression that these results should be interpreted as a test-retest-reliability indicator.

3) It is commendable that the authors collected longitudinal data in a routine care setting. However, the presentation of the sample size is somewhat misleading. While the authors report a total sample of 219 participants, only 140 and 108 individuals were available at the first and second follow-up, respectively. For the sake of transparency, I would prefer to speak of two sample subsets, e.g., a baseline sample (e.g., to examine discriminant and convergent construct validity) and the longitudinal sample set (e.g., to examine criterion validity).

- We thank the reviewer for this helpful suggestion. We have revised the manuscript to clarify the distinction between the baseline sample and the longitudinal subsamples. Specifically, we now describe the 219 patients who completed baseline measures as the **baseline sample**, while the 140 and 108 patients available at the first and second follow-ups, respectively, are referred to as the **longitudinal subsamples**. This change has been implemented in the
    - Abstract: p. 2, lines 16-18: *"The baseline sample comprised 219 patients with chronic pain; follow-up data were available from 140 participants at 3 weeks and 108 participants at 16 weeks, constituting the longitudinal subsamples."*
    - Methods p. 7, lines 25-26: "…based on the baseline sample for cross-sectional analyses and on the longitudinal subsamples for analyses involving follow-up data."
    - Results section (p. 8, lines 31-33) for greater transparency: "*At baseline, 219 patients completed the variables of interest, constituting the baseline sample. The longitudinal subsamples included 140 patients at the first follow-up and 108 at the second."*

- In addition, in line with the reviewer's comment, we acknowledge that the predictive analyses based on the longitudinal subsamples may be regarded as indicators of **criterion validity** (specifically predictive validity).
- As mentioned in response to comment 1, we have clarified in the Method section which dataset was used to assess which type of validity, p. 7 and 8: *"**Validity** To evaluate the validity of the GEEE, we conducted correlation and regression analyses based on the baseline and longitudinal samples. Analyses of the baseline dataset examined aspects of construct validity: intercorrelations among the GEEE expectation items assessed discriminant (improvement vs. worsening) and convergent validity (worsening vs. side effects), while correlations with depression tested discriminant validity. Associations between improvement expectations and desire for pain relief, analysed at all three time points, served as indicators of construct validity, as these two theoretically related constructs were expected to show a positive association.. Analyses of the longitudinal subsets (T2 and T3) addressed item validity, through correlations between GEEE treatment effect items and changes in clinical outcomes (pain intensity, PDI), and criterion validity, specifically predictive validity, through regression analyses testing whether baseline expectations predicted outcomes at follow-up.*"
- Additionally, we have added a sentence to the Discussion (p.13 lines 33-34): *"Improvement expectations predicting later symptom reduction may further be regarded as evidence of the GEEE's criterion validity, specifically its predictive validity."*

4) The presentation of the GEEE was confusing to me. I have also looked at the citations provided, but I still have some unanswered questions. As I understand it, the core items of the GEEE are the ones about improvement, worsening, and side effects expectations. But if I want to use this questionnaire in research or practice, what do I do with these items? Do I score them individually? Or in combination with past or current experience? Am I looking at change scores?
In this study, things seem to have been even more complicated because different treatments were rated separately. Did all patients rate all treatments? Or only the ones that were recommended to them? Were the expectation items then averaged or looked at again separately? Including a clearer description, possibly supported by a visual overview or table of the assessed items, would be very helpful.

- We thank the reviewer for this thoughtful comment and agree that the description of the GEEE and its application in our study required clarification. We have therefore revised the Methods section to provide a clearer account of the instrument, specifying that the three core items (improvement, worsening, side effects) were analyzed as individual average scores per item, not composite or change scores. We also clarified that only patients who had prior experience with a given treatment type (medication, physiotherapy, psychological treatment) responded to the corresponding previous experience items (i.e. prior improvement, worsening or side effect), while the treatment expectation and treatment effect items always referred to the pain treatment that patients would receive at the pain center in general, independent of specific modalities. Furthermore, we have specified the timing of assessments for each subscale. In addition, we have revised the corresponding Results section to clarify the interpretation of the associations between previous experiences and current expectations. These changes should improve transparency and make the use of the GEEE in this study clearer for readers.
- We have rewritten the description of the GEEE in the methods section (p. 6 and 7): *"**Treatment Expectations.** The Generic Rating Scale for Previous Treatment Experiences, Treatment Expectations, and Treatment Effects (GEEE) was used to assess three domains: previous treatment experiences, current treatment expectations, and current treatment effects with regard to pain treatment at the pain center (23). Each domain comprises three items (improvement, worsening, side effects), rated on an 11-point scale (e.g., 0 = 'no improvement,' 10 = 'greatest*

improvement imaginable') (Appendix D). Items were analysed individually, using average scores. The subscale on previous treatment experiences was divided into medication, physiotherapy, and psychological treatment; only patients who had had previous experience with the respective treatment provided ratings for that modality. By contrast, the subscales on current expectations and treatment effects referred to pain treatment at the center in genreal (i.e., "How much improvement in your symptoms do you expect from the pain treatment?"), independent of the specific modalities recommended or received. Expectation items were assessed at all time points, the previous experience subscale at baseline (T1), and the treatment effects subscale at the two follow-ups (T2, T3)."

- We also clarified this point in the results section (p.10, lines 20-23): "*Prior experiences of pain worsening from medication, physical therapy, or psychological treatments were associated with stronger expectations of worsening for the upcoming treatment (medication experience: Rho(125)=.411, p<.001; physiotherapy experience: Rho(143)=.346, p<.001; psychological treatment experience: Rho(53)=.414, p=.002; see Appendix B).*"
- Additionally, we now provide the GEEE items used in this study in Appendix D.

5) In my opinion, it would be better to limit the statistical analyses depending on the focus of the paper. It is also concerning that the authors apply a variety of statistical tests without giving any thought to correcting for alpha errors.

- We thank the reviewer for the valuable suggestion regarding limiting statistical analyses. As noted above, we have clarified throughout the manuscript which statistical analyses correspond to each of the two study aims and which samples were used for each analysis (baseline sample vs. longitudinal subsets). This clarification improves transparency and helps readers clearly follow the rationale behind each analysis. We have retained all statistical analyses because each contributes to addressing one of the two predefined study aims: (1) validating the GEEE (examining its psychometric properties and validity indicators) and (2) exploring the role of treatment expectations over time in a clinical setting, including their predictive value for treatment outcomes. We believe that maintaining the full set of analyses provides a comprehensive picture of the measure's validity and clinical utility.
- We also removed the correlations that did not directly serve the two aims (i.e., correlations between desire for pain relief with measures other than the expectation items).
- We also thank the reviewer for the important remark about correcting for alpha errors. It is an absolutely important point, and we fully agree with the reviewer and we can confirm that we have applied appropriate corrections for multiple testing. We thank the reviewer for drawing our attention to this and for giving us the opportunity to clarify and explicitly include this information in the manuscript. First, we would like to clarify that we used Tukey's correction to control for multiple comparisons in the post-hoc analyses of the repeated measures ANOVAs, although this was not explicitly stated in the original manuscript. Tukey's post-hoc correction was used because it is appropriate for pairwise comparisons following a repeated-measures ANOVA. It controls the familywise error rate while maintaining good statistical power for multiple comparisons between time points.
  - We added this important information to the Methods section (p. 8, lines 12- 13): "To evaluate changes in treatment expectations (improvement, worsening, and side effects) and clinical outcomes (pain-related disability, pain intensity), we conducted repeated measures ANOVAs with one within-subjects factor (time: baseline, T2, and T3), *followed by Tukey-corrected post-hoc comparisons.*"
- To further address the concern regarding alpha error correction, we have now additionally applied a Benjamini–Hochberg false discovery rate (FDR) correction to the regression analyses in order to control for multiple comparisons. We chose this method because it offers a good balance between limiting false positives and

maintaining statistical power, which is particularly appropriate in exploratory analyses with multiple but related outcomes.

    - This has been clarified in the Methods section (p.8, lines 16-17): "To test whether baseline expectations, as measured with the GEEE predict clinical outcomes (pain-related disability; pain intensity; GEEE: current improvement, worsening, and side effects) at the two follow-ups, we performed multiple regression analyses for each outcome *with Benjamini-Hochberg correction* at each follow-up.
    - and in Appendix A: "*Given that several regression analyses were conducted across related outcomes, we applied a false discovery rate (FDR) correction on the main analyses using the Benjamini–Hochberg procedure to control for multiple comparisons.*"

- The Benjamini-Hochberg-corrected p-values have been updated throughout the manuscript and in Appendix C. All reported results remained significant with the correction.

6) The discussion highlights that improvement and worsening/side-effect expectations only moderately correlate, suggesting they should be assessed separately. However, only improvement expectations significantly predicted pain intensity and disability at follow-up, which somewhat weakens their point. I have also open questions about why it is important to measure worsening and side effect expectations separately. Moreover, I am curious whether it would not be more relevant to look at individual changes, i.e., whether a person's pain improves or worsens more than expected. I think there is still a lot of potential in the data.

- We thank the reviewer for this thoughtful comment. We regard the correlational and predictive findings as somewhat separate results, both worth reporting. As part of the validation results we found a weak negative correlation between improvement and worsening expectations, which supports the view that they are distinct dimensions rather than opposite ends of the same scale, where a strong negative correlation would be expected. We consider this important to report, as previous studies report similarly weak or no correlations and thus evidence for the two constructs being separate is accumulating.
- We also found a moderate correlation between expectations of worsening and expectations of side effects, indicating that while both reflect negative anticipations, they capture distinct aspects of treatment-related cognition. Other studies using the GEEE show that expectations of side effects may coexist with positive outcome expectations and can help to reinforce treatment effects (e.g., through supporting onset sensations), whereas expectations of worsening reflect a generally pessimistic outlook. Assessing them separately therefore allows for a more nuanced understanding of negative treatment expectations and their potential impact on outcomes, as discussed on p. 14 of the manuscript. Nevertheless, further studies in diverse samples and clinical conditions are needed to evaluate the usefulness of these items for predicting treatment outcomes.
- It is true that in the exploratory analyses on the prediction of clinical outcomes, only improvement expectations predicted pain intensity and pain-related disability at follow-up, whereas worsening expectations did not. However, this may in part reflect floor effects, as patients in this sample reported very low baseline levels of worsening and side-effect expectations, likely limiting their predictive value. This may be due to the characteristics of our specific sample rather than a limitation of the GEEE itself. Patients often wait a long time for treatment at the center, likely resulting in very low scores for negative expectations and side effects due to a high need for treatment/desire for relief and accompanying positive treatment expectations. We suspect however, that in other clinical settings, where worsening and side effects expectations may be stronger, these domains could prove more predictive. We have noted the limitation in the manuscript in the strengths and limitations section.
- We also agree with the reviewer that examining whether outcomes meet or diverge from individual expectations would be highly valuable, but this lies beyond the

scope of the present paper. We have therefore incorporated it into the limitations section as an important avenue for future research.

- Strengths and limitations p. 15, lines 30-33: *"In this sample, low expectations of worsening and side effects suggest a floor effect that may have limited their predictive value. Further studies in diverse samples are needed to examine whether outcomes meet or diverge from patients' initial expectations and to evaluate the usefulness of these items for predicting treatment outcomes."*
- With respect to the reviewer's first point in the comment we have also slightly adapted the wording in the description and discussion of this result to clarify why the items should be measured separately, p.14, lines 1-4: "Interestingly, our data showed only a weak negative correlation between expectations of improvement and expectations of worsening, supporting the notion that these are two distinct dimensions *rather than two ends of the same scale. This aligns with previous mixed findings, with one study reporting a weak negative correlation in patients with endometriosis (26) and others finding no significant relationship in patients with chronic pain (25) and in diverse clinical samples (37). Overall, these findings highlight the importance of assessing positive and negative expectations separately."*

7) The limitations section refers to the dynamic nature of treatment expectations, which contradicts the statement of the paper that expectations were seemingly stable in this sample. This apparent contradiction is not addressed and could confuse readers. It may be advisable to either reconcile these points or consider omitting the stability analysis altogether, as mentioned earlier.

- We thank the reviewer for this comment. We have now clarified in the manuscript that the apparent stability of expectations in our results refers to relative stability at the group level across the three time points. At the same time, smaller within-person fluctuations may have occurred between assessments but could not be captured given the long intervals in our design. We have revised the limitations section accordingly to reconcile these points and to highlight that methods such as ecological momentary assessment would be better suited to investigate the dynamic nature of expectations.
- P. 16, lines 3-7: *"Fourth, although expectations are increasingly recognized as dynamic (44) and our repeated measurements represent a step toward understanding changes over time, our results indicated relative stability across the three time points. Smaller within-person fluctuations may nevertheless have occurred but were not detectable with our design. Future studies could apply ecological momentary assessment to examine such fine-grained dynamics."*

Reviewer: 2
Dr. Eleonora Maria Camerone, University of Oxford
Comments to the Author:
Please see my comments in the uploaded file.

Longitudinal assessment and clinical implications of treatment expectations in routine clinical settings: evaluation of the GEEE in patients with chronic pain.

This is an exciting and well-conducted study that seeks to bridge the gap between evidence on the powerful effects of treatment expectations from clinical trials and experimental research and their translation into real-world clinical practice. To this end, the authors explore the use of a new tool to measure treatment expectations (GEEE) in a pain management setting among patients seeking pain treatment (i.e., examining various correlations and stability over time), while also implementing a longitudinal design to test whether baseline treatment expectations predict later clinical outcomes. The study design is generally well-conceived, and the writing is clear and concise throughout. However, I have a few major and some minor comments that I recommend addressing to increase the rigour of the study.

Abstract.

1. *Line11(page3).* Specify the type of outpatients(e.g., pain sufferers or patients seeking pain treatment). I would not say chronic pain patients, because this is not the case for the whole sample.

    - We thank the reviewer for this valuable comment. While only 74.9% of participants had a formal ICD-10 diagnosis of chronic pain, we strongly assume that the remaining patients also experienced chronic pain, even if they had not yet formally received the corresponding diagnosis at baseline. Because the pain center is a tertiary referral pain clinic with long waiting times, it is highly likely that patients referred there had been experiencing pain for more than six months. For this reason we kept the sample description as patients with chronic pain and added an explanation in the manuscript (See responses to comments 9 and 13 for further details).

2. *Line9(page2). "The present study examines the use of this new instrument..."*- The term *examines* is rather unspecific. If the main aim of the study is to validate the GEEE scale (which remains unclear throughout the manuscript but becomes clearer in the Discussion, where validation appears to be a central aim), I would recommend stating this explicitly from the outset and using the term *validate* rather than *explore*.
    - We thank the reviewer for this helpful suggestion. In line with this comment and the comment of reviewer 1 on this issue, we have thoroughly revised the manuscript to clarify the two main aims of the study—(1) validation of the GEEE and (2) exploration of treatment expectations in a longitudinal, real-world clinical setting. Accordingly, we now use the term *validate* when referring to the first aim and reserve the term *explore* exclusively for the second, exploratory aim. Revisions were made in the abstract, introduction, methods, results and discussion to make this distinction more explicit:
        1. Abstract p. 2 lines 10-12: *"The present study aims to test the validity and applicability of this new instrument in patients seeking pain treatment. An additional exploratory aim is to assess the role of treatment expectations over time and to examine whether the GEEE is suitable for predicting treatment outcome longitudinally in this clinical setting."*
        2. Introduction p.5 lines 7-14: "*The aim of the present study is two-fold: (1) to validate the GEEE in a real-world clinical setting, and (2) explore the role of treatment expectations over time, including their value in predicting clinical treatment outcomes. To address the first aim, we examined relationships among GEEE-measured expectations (improvement, worsening, side effects) and other pain-relevant measures to evaluate aspects of construct validity. To address the second, exploratory aim, we assessed associations between previous treatment experiences and pre-treatment expectations, explored changes in expectations and clinical outcomes during treatment, and tested whether baseline expectations predicted later clinical outcomes."*
        3. Methods, Statistical analysis p. 7-8: *"**Validity** To evaluate the validity of the GEEE, we conducted correlation and regression analyses based on the baseline and longitudinal samples. Analyses of the baseline dataset were used to examine aspects of construct validity: intercorrelations among the GEEE expectation items assessed discriminant (improvement vs. worsening) and convergent validity (worsening vs. side effects), while correlations with depression tested discriminant validity. Associations between improvement expectations and desire for pain relief, analysed at all three time points, served as further indicators of construct validity, as these two theoretically related constructs were expected to show a positive association.. Analyses of the longitudinal subsets addressed item validity, through correlations between GEEE treatment effect items and changes in clinical outcomes (pain intensity, PDI), and criterion validity, specifically*

predictive validity, through regression analyses testing whether baseline expectations predicted outcomes at follow-up.
Treatment Expectations over Time *To explore the longitudinal role of treatment expectations, we first examined the associations between baseline expectations and previous treatment experiences (medication, physical therapy, and psychological treatments) using Spearman rank correlations. These analyses aimed to assess whether prior treatment experiences were related to patients' initial expectations of improvement, worsening, or side effects. To evaluate changes in treatment expectations (improvement, worsening, and side effects) and clinical outcomes (pain-related disability, pain intensity), we conducted repeated measures ANOVAs with one within-subjects factor (time: baseline, T2, and T3), followed by Tukey-corrected post-hoc comparisons. To test whether baseline expectations, as measured with the GEEE predict clinical outcomes (pain-related disability; pain intensity; GEEE: current improvement, worsening, and side effects) at the two follow-ups, we performed multiple regression analyses for each outcome with Benjamini-Hochberg correction at each follow-up. In each regression 11 predictors (age, gender, baseline anxiety and depression, GEEE expectations, incapacity for work, desire for pain relief, and baseline pain intensity and pain-related disability) were entered as independent variables in one step. Post-hoc power analyses were conducted for all regression models (36). (See Appendix A for additional information.)"*

4. Results section on p.9 and p.10 is now divided into results relating to the validity aim (p.9) and the exploratory aim (role of treatment expectations over time on p. 10)
5. Discussion p.13, lines 9-13: *" In this study, we investigated the validity and clinical utility of the novel generic assessment of expectation scale GEEE in chronic pain patients of a routine pain care center. We found evidence supporting the validity of the GEEE."*
6. Discussion p. 13, lines 22-27: "*At baseline, inter-item correlations provided preliminary internal evidence of discriminant (improvement vs. worsening/side-effect) and convergent validity (worsening vs. side-effect), while the absence of associations with depression further supported discriminant validity. The GEEE's improvement expectation item consistently correlated with desire for pain relief, providing supportive evidence of construct validity, acknowledging that the two constructs are theoretically related but not identical."*

Introduction.

3. *Line10(page4).*The term 'placebo analgesia' appears here for the first time; I suggest adding a brief definition and considering the more accurate term 'placebo hypoalgesia,' as it usually refers to reduced - not eliminated - pain.
    - Thank you for this valuable comment. We have now replaced 'placebo analgesia' with the more precise term of placebo hypoalgesia and added a short definition:
        - Introduction p. 4, lines 10-11: "Expectations seem to not merely alter subjective experiences, but can also influence endogenous physiological processes – such as the release of endogenous opioids in the case of placebo *hypoalgesia (i.e., Reduced pain perception induced by expectation, without an active pharmacological treatment)."*

Statistical Analyses.

4. How was missing data managed? Please specify.

    - We thank the reviewer for the opportunity to clarify this point. Missing data were managed using complete case analyses (as stated on p. 7, lines 25-26), such that only participants with complete data on the variables of interest were included in each analysis. In response to a comment by reviewer 1, we have changed the

wording around the sample composition and now refer to a "baseline sample" and the longitudinal subsets. For the baseline sample, analyses included all participants with complete baseline data, while longitudinal analyses were conducted on the longitudinal subsamples, consisting of participants with data available across the relevant time points. While this approach may reduce sample size and limit statistical power, it ensures that the analyses are based solely on observed data without the additional assumptions required for data imputation (e.g., that data are missing at random).

- To address limitations of this approach for the regressions, we have added a corresponding statement to the *Strengths and Limitations* section.
  - Strengths and Limitations p. 16, lines 10-12: *"Moreover, because the regression analyses at T3 were based on a reduced sample with multiple predictors, the risk of model overfitting cannot be ruled out."*
- Additionally, and in response to comment 4 of Reviewer 3, we have now also conducted linear mixed model (LMM) analyses to account for missing data and verify the robustness of our repeated-measures ANOVAs findings. The results of the LMM analyses were consistent with those of the repeated-measures ANOVAs and are reported in Appendix E.

5. Sample size calculation. I could not find a description of the sample size calculation. Although the sample size is relatively large (n = 219), one of the primary aims is to predict clinical outcomes from baseline treatment expectations. Prediction models often require large samples to ensure adequate statistical power, and adequacy depends on the number of predictors. My understanding is that there are at least eight (more likely 10) predictors: age, gender, baseline DASS, GEEE expectations (potentially three predictors given the subscales), incapacity for work, desire for pain relief, and baseline pain intensity and pain-related disability. I suggest making the number of predictors explicit. If no a priori sample size calculation was performed, please consider adding a post hoc power analysis or explaining how the sample size was determined.

- We thank the reviewer for this important comment. We added an explanation in the statistical analysis section to make this point more transparent (p.7 lines 22-24): *"As this study utilized a consecutive clinical sample, no a priori sample size calculation was conducted. The available sample size was determined by the number of eligible patients during the study period, rather than by power considerations for the present analyses."*
- We have also made the number of predictors explicit in the Method section, p. 8 line 17: "In each regression *11 predictors* (age, gender, baseline anxiety and depression, GEEE expectations, incapacity for work, desire for pain relief, and baseline pain intensity and pain-related disability) were entered as independent variables in one step.
- Following your suggestion, we also added a post-hoc power analysis for the regression models and added the results of the post-hoc power calculations in Appendix A (additional statistical information): "*Post-hoc power analyses indicated that the regression models predicting GEEE subjective improvement, pain intensity, and PDI were well-powered ($1 - \beta$ = .96–1.00). In contrast, the models predicting GEEE subjective worsening and side effects showed more variable power ($1 - \beta$ = .41–.97).*"

6. Correlation Analyses. *Lines 27-28 (page 7).* The authors mention testing the correlation between the GEEE improvement expectation item and 'desire for pain relief' to assess convergent validity. However, I am not convinced by this approach, as I do not think the two measures are assessing the same construct. While these variables may or may not be correlated (and it is great that the authors test this), wanting pain relief (desire for pain relief) is not the same as expecting pain relief. In fact, previous experiences might influence these differently: for example, one might have very low expectations of pain relief while still having a strong desire to experience less pain. Please let me know if I am missing something here.
   - We thank the reviewer for this valuable comment. We agree that *expectation of pain relief* and *desire for pain relief* are conceptually distinct constructs.

Expectation of pain relief primarily reflects a cognitive, probabilistic, appraisal of anticipated outcomes, whereas desire for pain relief captures the affective motivation to achieve relief. Our rationale for including the correlation with 'desire for pain relief' was that both variables reflect patients' treatment-related attitudes, and we anticipated at least a moderate association. However, we acknowledge that this represents a relatively weak indicator of convergent validity and may be an indicator of construct validity more broadly, as the two constructs are distinct, yet theoretically related. We have therefore revised the manuscript to clarify our reasoning and to highlight this conceptual distinction.

- Method section (p. 7, lines 34-36): *"Associations between improvement expectations and desire for pain relief, analysed at all three time points, served as further indicators of construct validity, as these two theoretically related constructs were expected to show a positive association."*
- Discussion (p.13, lines 24-27): "*The GEEE's improvement expectation item consistently correlated with desire for pain relief, providing supportive evidence of construct validity, acknowledging that the two constructs are theoretically related but not identical.*"

7. Correlation Analyses. *Line 28-29 (page 7).* The authors write: "*we assessed correlations (v) between GEEE current treatment effects and changes in clinical outcomes*". Could the authors clarify (1) why these correlations were run (e.g., as part of the GEEE validation strategy?) and (2) the exact time points involved? My understanding is that these are associations at each follow-up (T2, T3) between current perceived improvement, worsening, and side effects and changes from baseline (correct?). I suggest stating this explicitly in the Methods and framing them as validation analyses (if that is the case) to distinguish them from the predictive regression models.
   - We thank the reviewer for this helpful comment. We have carefully revised the manuscript to more clearly distinguish between the two aims of the study and to specify which analyses correspond to each aim.
   - In the Method section (p.9/10) we have added sections for the two aims detailing which analysis, corresponded to which aim.
   - We added the suggested details about the assessment time points and the reason for running these correlations (item validation of the GEEE current treatment effects items).
     - Method section (Validity, p. 7, line 36 and 37, p.8 lines 1-3): *"Analyses of the longitudinal subsets addressed item validity, through correlations between GEEE treatment effect items and changes in clinical outcomes (pain intensity, PDI), and criterion validity, specifically predictive validity, through regression analyses testing whether baseline expectations predicted outcomes at follow-up."*
8. Has the study been pre-registered?
   - The present study was not pre-registered as a standalone project. However, the broader clinical data collection, from which the current dataset was derived, is registered in the German Clinical Trials Register (DRKS00022951, https://drks.de/search/de/trial/DRKS00022951). This registry entry covers the standardized assessment of psychological and clinical variables in pain patients, including treatment expectations. At the time of registration, treatment expectations were assessed using the *Credibility/Expectancy Questionnaire* (Devilly & Borkovec, 2000). Once the GEEE became available, it replaced that measure in the standardized assessment battery used in this clinical cohort.
   - We have added the information on the trial registration in the declarations section of the manuscript (p.17): *"**Trial registration**: The broader data collection from which this dataset was derived is registered in the German Clinical Trials Register (DRKS00022951). At the time of registration, treatment expectations were assessed using the Credibility/Expectancy Questionnaire (Devilly & Borkovec, 2000); this measure was later replaced by the GEEE when it became available."*

Results.

9. Demographic Characteristics. *Line 9 (page 8).* Please specify how *chronic pain* was defined (e.g., pain persisting for longer than six months). Additionally, as only 75% of patients had chronic pain, could the authors clarify how the remaining participants were classified (e.g., acute pain)? I suggest adding details on eligibility criteria for participants in the methods section under the 'Participant' subsection (page 5).
    - We thank the reviewer for this helpful comment. We have added a clarification in the Results section to specify how chronic pain was defined and to describe the composition of our sample in more detail. Most patients (75%) were diagnosed with chronic pain according to ICD-10 criteria (e.g., F45.41, pain persisting for more than six months). As the pain center is a tertiary referral clinic with long waiting times, it is likely that the remaining patients also suffered from chronic pain, although not yet formally diagnosed at baseline.
    - The revised sentence (p. 8, lines 26-28) now reads: *"Overall, 75% of the sample had a chronic pain diagnosis (F45.41, pain persisting for more than six months) at baseline, while the remaining patients were referred as chronic pain patients without official diagnosis."*
10. Demographic Characteristics. *Lines 10-11 (page 8).* It is great that the authors reported prior experience with the pain treatment modalities of focus for this study (i.e., medication, physical treatment, and psychological treatment). However, it would also be informative to know whether patients had tried other types of treatments. Have the authors assessed this? If so, it would be helpful to include data such as the total number of pain treatments tried by patients.
    - We thank the reviewer for this excellent suggestion. We agree that information on the total number and broader range of pain treatments would provide important additional context. Unfortunately, such data were not collected in the present study. We will ensure to include this in future studies to allow for a more comprehensive characterization of participants' treatment histories.
11. Correlation Analyses - GEEE Expectation *(page 8).* The rationale for conducting correlation analyses between GEEE expectation items at baseline is unclear. In my view, these analyses could represent a reasonable approach to assessing item validity. Could the authors clarify whether this was indeed their rationale? If so, I would recommend stating this explicitly in the text, as was done for the correlation between baseline expectation items and depression scores. Specifically, testing the correlation between improvement expectations and expectations of worsening appears to be a reasonable way to assess discriminant validity (please feel free to disagree). Similarly, the correlation between expectations of worsening and expectations of side effects seems a reasonable way to assess convergent validity, as both reflect negative treatment outcome expectations. Clarifying these points would, in my view, strengthen the manuscript and further advance the validation of the GEEE.
    - We thank the reviewer for this thoughtful comment and fully agree that these correlations provide meaningful insights into the validity of the GEEE. In response to this and related comments by the other reviewers, we have now revised the manuscript throughout to clarify the study's dual aims (validation and longitudinal exploration) and to specify which analyses were conducted for each aim (p. 7, from line 30). In particular, we now explicitly state in the Methods section that the correlations between the GEEE expectation items at baseline were conducted as part of the validation strategy, with the correlation between improvement and worsening expectations reflecting discriminant validity and that between worsening and side-effect expectations reflecting convergent validity. We hope these clarifications resolve the reviewer's concern and enhance the transparency of the rationale and analytical approach.
    - We agree that the correlations between the GEEE expectation items can be meaningfully interpreted in terms of validity. We have added and clarified this rationale throughout the manuscript:
        - Methods section (p.7 lines 30-34) as suggested: "*To evaluate the validity of the GEEE, we conducted correlation and regression analyses based on the baseline and longitudinal samples. Analyses of the baseline dataset examined aspects of construct validity: intercorrelations among the GEEE expectation items assessed discriminant (improvement vs. worsening) and*

convergent validity (worsening vs. side effects), while correlations with depression tested discriminant validity."
  - Results section (p.9 lines 13-16): "At baseline, improvement expectations showed small negative correlations with expectations of worsening (Rho(217)=-0.15, *p*=.028) and side effects (Rho(217)=-.14, *p*=.034), *supporting discriminant validity*. Additionally, expectations of worsening and of side effects were positively associated with one another (Rho(217)=.31, *p*<.001), *supporting convergent validity.*
  - Discussion (p.13, lines 22-24): *"At baseline, inter-item correlations provided preliminary internal evidence of discriminant (improvement vs. worsening/side-effect) and convergent validity (worsening vs. side-effect), while the absence of associations with depression further supported discriminant validity."*

12. Repeated Measures ANOVAs – Pain Intensity and Pain-Related Disability. *Line 55 (page 9) and line 5 (page 10).* Were the post-hoc analyses corrected for multiple comparisons? Please specify.
    - Thank you for this important remark. Yes, we have used Tukey's correction to control for multiple comparisons in the post-hoc analyses.
      - We added this important clarification to the Methods section (p.8, line 12): "To assess whether expectations as measured by the GEEE (improvement, worsening, and side effects) and clinical outcomes (pain-related disability, pain intensity) change over time, we performed separate repeated measures ANOVAs with one within-subjects factor (time: baseline, T2, and T3), *followed by Tukey-corrected post-hoc comparisons."*
    - In addition, we have now applied a Benjamini–Hochberg false discovery rate (FDR) correction to the regression analyses in order to control for multiple comparisons. We chose this method because it offers a good balance between limiting false positives and maintaining statistical power, which is particularly appropriate in exploratory analyses with multiple but related outcomes.
      - This has been clarified in the Methods section (p.8, lines 16): "To assess predictors of the clinical outcomes (pain-related disability; pain intensity; GEEE: current improvement, worsening, and side effects) at the two follow-ups, we performed one multiple regression analysis per outcome *with Benjamini-Hochberg correction at each follow-up."*
      - and in Appendix A: "*Given that several regression analyses were conducted across related outcomes, we applied a false discovery rate (FDR) correction on the main analyses using the Benjamini–Hochberg procedure to control for multiple comparisons."*
    - The Benjamini-Hochberg-corrected p-values have been updated throughout the manuscript and in Appendix C.

Discussion – Overall, it's concise, well-structured, and truly exciting.

13. *Line 14 (page 12).* The statement *"in a sample of patients with chronic pain"* seems inaccurate, as only 74.89% of participants suffer from chronic pain. Given the established role of expectations in chronic pain (e.g., inflexible expectations), the inclusion of a hybrid sample with both chronic and more acute (?) pain patients is an important limitation. I recommend clarifying in the Discussion (and in the limitations section) that the sample is mixed, explaining why non-chronic patients were included, and considering how this may affect interpretation. One possible - though sample-size–limiting - approach would be to re-run analyses excluding non-chronic patients.
    - We thank the reviewer for this valuable comment. While only 74.9% of participants had a formal ICD-10 diagnosis of chronic pain, we strongly assume that the remaining patients also experienced chronic pain, even if they had not yet received the corresponding diagnosis at baseline. Because the pain center is a tertiary referral pain clinic with long waiting times, it is highly likely that patients referred there had been experiencing pain for more than six months.
    - As noted, the sample included both mostly chronic patients with a diagnosis, yet we cannot exclude the possibility that patients with more acute pain were included in the sample. This sample was used because our aim was to assess the

psychometric properties of the GEEE in a naturalistic outpatient setting, reflecting the heterogeneity typically expected in clinical practice. We did not re-run the analyses without the potentially non-chronic patients, as these patients most likely also suffered from chronic pain, which was just not formally diagnosed at baseline yet, and retaining them ensured a realistic representation of our clinical sample.

- Following the reviewer's suggestion, we have clarified the sample description in the Results section and added a statement to the *Strengths and Limitations* section noting that the potential inclusion of patients without an official diagnosis may limit generalizability. However, we also highlight that this reflects real-world clinical practice in such a setting and thus enhances the external validity of our findings.
    - Results p.8 lines 26-28: *"Overall, 75% of the sample had a chronic pain diagnosis (F45.41, pain persisting for more than six months) at baseline, while the remaining patients were referred as chronic pain patients without official diagnosis."*
    - Strengths and Limitations (p.15, lines 26-30): *"A limitation of this study is that the sample consisted of patients with and without formal chronic pain diagnosis. While this may limit generalizability, it reflects real-world clinical conditions in tertiary pain care and thus enhances external validity. Future studies could distinguish between chronic and acute pain patients to clarify potential differences in expectation effects.."*

14. *Lines 26-27 (page 12).* Not sure I agree with the following statement: "*The GEEE's improvement expectation item showed significant correlations with desire for pain relief across all time points, indicating convergent validity".* See comment 6 regarding correlation analyses and convergent validity (corr. GEEE improvement expectation item and 'desire for pain relief).
    - We thank the reviewer for this valuable comment. As noted in our response to comment 6, we agree that *expectation of pain relief* and *desire for pain relief* represent conceptually distinct constructs, with the former reflecting a more cognitive component and the latter an affective one. Accordingly, we have revised the manuscript to clarify that these associations were examined as an indicator of *construct validity* rather than *convergent validity*. This change has been implemented consistently throughout the manuscript.
    - In response to the sentence the reviewer refers to in this comment, we have changed it to: "The GEEE's improvement expectation item consistently correlated with desire for pain relief, *providing supportive evidence of construct validity, acknowledging that the two constructs are theoretically related but not identical."*(p.13, lines 24-27)
    - Please also see our responses to comment 6.

15. As indicated in the 'Validity' section of the Discussion *(page 12)*, one of the study's aims is to, at least in part, validate the GEEE instrument. I suggest making this explicit from the outset, as it is currently only partially clear.
    - We thank the reviewer for this helpful comment. As noted in our response to comment 2 and also reviewer 1, we have revised the manuscript throughout to make the dual aims of the study explicit—namely, (1) the validation of the GEEE and (2) the exploration of treatment expectations in a longitudinal, real-world clinical setting. The structure and phrasing of the abstract, introduction, and methods have been adjusted accordingly to ensure that the validation objective is clearly stated from the outset.

16. When presenting the prediction results (page 13-14), the authors report only the significant findings at the 16-week follow-up, without discussing why the results differ at the 3-week follow-up (where only pain intensity/pain-related disability emerged as a significant predictor). It would be valuable to add a brief comment on the potential role of temporal expectations and to explore why the 3- and 6- week outcomes differ. One possible explanation is that patients may not expect to improve shortly after treatment (e.g., at 3 weeks), but may anticipate treatment effects to manifest later (e.g., at 16 weeks). The following studies on temporal expectations may be relevant:

- Camerone, E. M., Piedimonte, A., Testa, M., Wiech, K., Vase, L., Zamfira, D. A., ... & Carlino, E. (2021). The effect of temporal information on placebo analgesia and nocebo hyperalgesia. *Psychosomatic Medicine*, *83*(1), 43-50.
- Camerone, E. M., Wiech, K., Benedetti, F., Carlino, E., Job, M., Scafoglieri, A., & Testa, M. (2021). 'External timing'of placebo analgesia in an experimental model of sustained pain. *European Journal of Pain*, *25*(6), 1303-1315.

Response:

- We thank the reviewer for this helpful suggestion. We also believe that the communication around when treatment effects can be expected play an important role in clinical practice. In the revised manuscript, we now explicitly note that improvement expectations predicted treatment outcomes at the 16-week follow-up but not at the 3-week follow-up. To address the reviewer's point, we have now added a sentence discussing the potential role of temporal expectations, highlighting that prior information by the clinical team typically emphasize longer timescales of treatments and life-style changes to unfold their action which may have shaped patients' symptom trajectories. We have now cited Camerone et al. (2021a, *Psychosomatic Medicine*), Camerone et al. (2021b, *European Journal of Pain*), and Valeria et al. (2025, *European Journal of Pain*), who provide converging experimental and neurobiological evidence that verbal information about timing can modulate the onset of placebo analgesia.
    - The addition can be found in the Discussion (p. 15, lines: 1-6): *"*Notably, improvement expectations consistently predicted symptom reduction across the different outcomes at the 16-week follow-up. *However, improvement expectations did not predict outcomes at the 3-week follow-up. One possible explanation is that several interventions need more time to show strong effects (e.g., treatment with antidepressants, physical therapy, psychological treatments). Further, verbal suggestions about when treatment effects would occur may have shaped patients' temporal expectations, such that improvements were anticipated later rather than earlier (49–51)."*

Reviewer: 3
Dr. Helen Purtill, University of Limerick
Comments to the Author:
This study presents an interesting research topic in the evaluation and use of GEEE in patients with chronic pain.

1. Missing data (lost to follow-up)
The study design is longitudinal across 3 timepoints (baseline, T2 (>3 weeks), T3 (>16 weeks) but suffers from a high dropout (n = 219 at baseline, n = 140 at T2, n = 108 at T3). It would be of importance to examine whether those who dropped out of the study differed across the measured baseline characteristics (demographic, pain intensity, disability etc) to those who remained in the study. As over 50% of participants were no longer in the study at T3 this really needs to be acknowledge in the limitations, with careful consideration about how this may have affected the results. The Strengths and Limitations section pg 2,3 currently makes no acknowledgement of the potential effects of missingness and lost to follow-up. For example, it could be that those who are more motivated and more expressive are more likely to remain in the study until T3.

- We thank the reviewer for this important point. We have now conducted dropout analyses comparing completers and non-completers on baseline characteristics. These analyses indicated no differences at T2. At T3, however, non-completers reported significantly higher baseline pain intensity, depression, anxiety, and desire for pain relief than completers, whereas gender did not differ significantly. This suggests that attrition may have biased the results toward patients with comparatively healthier baseline profiles in terms of pain intensity and mental health at baseline. We have added this limitation explicitly to the Strengths and Limitations section of the manuscript and clarified the changes in sample size in the abstract.

- Abstract, p.2, lines 16-18: "***Participants:*** "*The baseline sample comprised 219 patients with chronic pain; follow-up data were available from 140 participants at 3 weeks and 108 participants at 16 weeks, constituting the longitudinal subsamples".*
- Results, p. 9, lines 1-3: "*Dropout analyses indicated no baseline differences between completers and non-completers at T2. At T3, however, non-completers reported higher baseline pain intensity, depression, anxiety, and desire for pain relief (all p < .05).*"
- Discussion, Strengths and Limitations: p. 16, lines 8-10: "*Finally, attrition was substantial which is common in naturalistic longitudinal data collection; at T3, non-completers had higher baseline symptom severity in terms of pain intensity, depression and anxiety and desire for pain relief, indicating that this was a more seriously affected subgroup.*"

2. Presentation of Descriptive Statistics:
Table 1 should also include characteristics described at all timepoints (where they are measured) with sample sizes given in the column headings. At present there are no descriptives given for current treatment experiences at T2 and T3 or GEEE at T2 and T3. In Table 1 means and SDs are given to summarise the numeric data but where the data is skewed (for example Expectation worsening and Expectation side effects look to be positively skewed) it should be summarised as median (IQR) or median (25th percentile, 75th percentile). Expectation of side effects and expectation of worsening appears to be very low at baseline so there would be floor effects - it would not be possible for someone to have a value lower than 0.

- We thank the reviewer for these helpful suggestions regarding Table 1. We have now revised the table (p. 9) accordingly. Specifically, we now provide descriptive statistics for all relevant variables at baseline, T2 (3 weeks), and T3 (16 weeks), with sample sizes given in the column headings. For skewed variables (expectation of worsening and expectation of side effects), we report both the mean (SD) and the median (25th–75th percentile), which more accurately reflects their distributions. As expected, these variables showed strong floor effects, and we discuss this in the Strengths and Limitations section of the Discussion. The floor effects may be due to the characteristics of our specific sample rather than a limitation of the GEEE itself. Patients often wait a long time for treatment at the pain center, likely resulting in very low scores for negative expectations and side effects. We suspect however, that in other clinical settings, where worsening and side effects expectations may be stronger, these domains could prove more predictive. We have noted the limitation in the manuscript in the strengths and limitations section.

    - Discussion (p.15, lines 30-33): *"In this sample, low expectations of worsening and side effects suggest a floor effect that may have limited their predictive value. Further studies in diverse samples are needed to examine whether outcomes meet or diverge from patients' initial expectations and to evaluate the usefulness of these items for predicting treatment outcomes."*

- We have also updated Appendix A to list the variables identified as skewed at each timepoint, based on skewness values exceeding ±1.

3. Correlation Analyses:
Correlations are presented which examine the strength of the baseline linear associations between GEEE scales, GEEE and previous treatment (but no consideration of when the previous treatment occurred), GEEE and depression. It may also be interesting to examine if any demographic characteristics (age, sex, back pain), baseline pain intensity, disability are associated with baseline GEEE scales - i.e. are there any participant characteristics associated with higher expectations?

- We thank the reviewer for these suggestions. We agree that considering the timing of previous treatments would be a very interesting research question, as it could

further clarify how recent or distant treatment experiences shape current expectations. However, this level of temporal detail was beyond the scope of the present study, which focused on validating the GEEE and examining its associations within a clinical context.

  - We have, however, included the idea into the future directions section of the Discussion, p.16, lines 1-2: "…future research should adopt a more nuanced approach, incorporating additional measurement time points and separate assessments for various treatment types (e.g., pharmacological, psychological, physical therapy), as the treatment type might interact with patient preferences and decision-making. *The timing of previous treatment experiences may also be an important factor to consider."*

- Following the recommendation, we examined correlations between baseline GEEE expectation items and demographic (age, gender) as well as clinical variables (pain intensity, pain-related disability, incapacity to work, depression, anxiety). The expectation items were generally unrelated to these characteristics, except for side-effect expectations, which correlated positively with anxiety. We have added this information to the Results section and the link between anxiety and side effect expectations in the Discussion.
  - Results, p. 9 lines: 18-19: "None of the three expectation items correlated significantly with depression scores, indicating discriminant validity. *Only side-effect expectations correlated with anxiety (Rho(212)=.16, p=.02). All baseline expectations were unrelated to demographic characteristics."*
  - Discussion, p. 14 lines 11-12: *"Additionally, higher anxiety was associated with stronger side-effect expectations, also consistent with previous findings on medical interventions (41)."*

Pg 9. Line 23 - 24: "All of these results confirm the validity of the current treatment effect items of the GEEE.." - While correlation analysis may be consistent with or provide evidence for validity it can not alone cannot confirm the validity of a scale. Assessing the validity of a scale is a much broader task beyond the design and analyses in this study.

- We thank the reviewer for this valuable comment. We agree that correlation analyses alone cannot confirm the validity of a measure. We have therefore revised the wording in the manuscript to more accurately reflect that the findings *support* rather than *confirm* the validity of the current treatment effect items of the GEEE.
  - Abstract p.2 line 35-36: "*We found supporting evidence for the validity of the GEEE and its applicability in longitudinal clinical research."*
  - Results p. 10, line 16: "*All of these results provide supporting evidence for the validity of the current treatment effect items of the GEEE."*
  - Discussion: p.13, lines 9-11: *"In this study, we investigated the validity and clinical utility of the novel generic assessment of expectation scale GEEE in chronic pain patients of a routine pain care center. We found evidence supporting the validity of the GEEE."*
  - p.13, lines 19-22: "Our study *provides further support for the validity of the GEEE* as an instrument for measuring treatment expectations (over time), in line with previous research that linked the GEEE to established measures such as the Credibility/Expectancy Questionnaire and the Treatment Expectation Questionnaire (25,26)."
  - Discussion, Strengths and Limitations, p.15 lines 19-23: *"First, our study extends previous validation work by including longitudinal aspects in the assessment of the GEEE's validity in a clinical pain management context. However, as this investigation was based on a naturalistic clinical sample rather than a study designed exclusively for validation purposes, the evidence for validity must be interpreted within this context. A dedicated validation study, with more controlled conditions, could further substantiate these findings."*

4. Repeated Measures ANOVA of GEEE, pain intensity and disability

Repeated measures ANOVA provides a complete case analysis and the results of this analysis are only relevant to those who have completed all 3 timepoints (less than half of the 219 who started). A linear mixed model analysis would provide an analysis of all participants who provided at least one measurement and should be used instead of repeated measures ANOVA or the limitation of using complete case analysis should be clearly stated.

- We thank the reviewer for this important observation. We agree that repeated-measures ANOVAs rely on complete cases and therefore reflect only participants with data at all three time points. To address this, we have taken up the suggestion of re-running the analyses as linear mixed model analyses. The results of the analyses confirm the results of the repeated-measures ANOVAs. We now report the results of the linear mixed model analyses in the Results section and a detailed description of the results has been prepared in Appendix E.
    - Results, p. 10, lines 34-36: *"Linear mixed model analyses yielded the same pattern of results, confirming the findings from the complete case repeated-measures ANOVAs (see: Appendix E)."*
    - See Appendix E for a detailed account of the results.

5. Regression analyses: Assumptions of regression rely on the normality and constant variance of the residuals - this should be examined and reported on for the regression analyses.
Regression analysis of GEEE Current Treatment effects - the R-square for these models are very low reflecting low accuracy in any predictions for these outcomes.
Prediction of pain intensity and disability at times T2 and T3 provide results that are somewhat conflicting. At T2 only baseline pain predicts pain at T2, similarly for disability (only baseline disability predicts disability). But at T3 (when the sample size has reduced), Treatment expectation predicts pain, similarly for disability.
The sample size for these regression models at T3 is only 100 but there are 11 predictor variables, there is a risk of overfitting a regression model to a small sample.
The conclusions from these models need to be framed within an acknowledgement of a relatively small sample size with many predictor variables.

- We thank the reviewer for these detailed and constructive comments.
- We would like to confirm that we inspected the regression residuals using Q–Q and residual–fitted plots, which indicated no major deviations from normality or homoscedasticity. A corresponding statement has now been added to Appendix A Additional Statistical Information
    - *"Assumptions for the regression analyses were examined. Autocorrelation was tested using the Durbin–Watson statistic, and multicollinearity was assessed via variance inflation factors (VIFs). Visual inspection of Q–Q plots and residual plots indicated no major deviations from normality or homoscedasticity."*
- We agree that the R^2 values for the GEEE Current Treatment Effect models were only modest. We have acknowledged this in the Discussion
    - Discussion p. 15, lines 6-9: "Interestingly, expectations of worsening also predicted greater subjective improvement at the 16-week follow-up, perhaps reflecting a subgroup of positively surprised patients (45). *Given the modest explanatory power of this model, these findings should be interpreted with caution."*
- We thank the reviewer for the thoughtful comment about the difference in prediction results. We acknowledge that the sample size at T3 was relatively small, which increases the risk of model overfitting. To address this, we added a corresponding note to the limitations section.
    - Strengths and Limitations, p. 16, lines 10-12: "*Moreover, because the regression analyses at T3 were based on a reduced sample with multiple predictors, the risk of model overfitting cannot be ruled out."*
- Additionally we have added and reported post-hoc power analyses for the regressions, which indicated moderate to high statistical power, supporting the robustness of the main findings despite this limitation.

- Methods, p.8, lines 19-20: *"Post-hoc power analyses were conducted for all regression models (36)."*
  - Appendix A Additional statistical information: "***Power Analyses.*** *Post-hoc power analyses indicated that the regression models predicting GEEE subjective improvement, pain intensity, and PDI were well-powered (1 – β = .96–1.00). In contrast, the models predicting GEEE subjective worsening and side effects showed more variable power (1 – β = .41–.97)."*
- As also noted in response to Reviewer 2 (comment 16), the differing prediction patterns likely reflect temporal expectations: in this clinical context, patients can typically expect treatment effects unfold over weeks to months, aligning with the 16-week rather than the 3-week follow-up. The earlier time point was included for exploratory purposes, while the later one represents the clinically relevant expectation horizon. Conceptually, we discuss the differences between the T2 and T3 regression findings in the Discussion section, highlighting the interplay between treatment expectations and temporal dynamics of treatment effects as a plausible explanation for the observed pattern.
  - Discussion (p.15, lines 1-6): *"However, improvement expectations did not predict outcomes at the 3-week follow-up. One possible explanation is that several interventions need more time to show strong effects (e.g., treatment with antidepressants, physical therapy, psychological treatments). Further, verbal suggestions about when treatment effects would occur may have shaped patients' temporal expectations, such that improvements were anticipated later rather than earlier (49–51)."*

Other comments:

Pg 2: Line 15: "We utilized the GEEE as an economic tool to assess treatment expectations.." Needs to be rephrased as it reads like GEEE provides an economic assessment.

- We have slightly adjusted the Abstract and the wording about the use of the GEEE, the new version no longer contains this sentence.

Pg 2, Line 17: "Improvement expectations correlated with desire for pain relief, indicating convergent validity." - but convergent validity is where two scales are designed to measure the same concept - but surely expectation of improvement and a desire for pain relief are different concepts? Also, the correlation shows only that they are weakly correlated (rho ≅ 3.5).

- We thank the reviewer for this valuable comment. We agree that *expectation of pain relief* and *desire for pain relief* are conceptually distinct constructs. Expectation of pain relief primarily reflects a cognitive, probabilistic, appraisal of anticipated outcomes, whereas desire for pain relief captures the affective motivation to achieve relief. Our rationale for including the correlation with 'desire for pain relief' was that both variables reflect patients' treatment-related attitudes, and we anticipated at least a moderate association. However, we acknowledge that this represents a relatively weak indicator of convergent validity and may be an indicator of construct validity more broadly, as the two constructs are distinct, yet theoretically related. We have therefore revised the manuscript to clarify our reasoning and to highlight this conceptual distinction.
  - Method section (p. 7, lines 34-36): *"Associations between improvement expectations and desire for pain relief, analysed at all three time points, served as further indicators of construct validity, as these two theoretically related constructs were expected to show a positive association."*
  - Discussion (p.13, lines 24-27): "*The GEEE's improvement expectation item consistently correlated with desire for pain relief, providing supportive evidence of construct validity, acknowledging that the two constructs are theoretically related but not identical."*

Pg 8, Line 22 - 23: "....Improvement expectations showed modest negative correlations with expectations of....", the rho values show small/weak correlations of -0.15 and -0.14 - these would not be considered modest.

- We have changed the wording to "*small* negative correlations." (p. 9, line 13)

VERSION 2 - REVIEW

**Reviewer**	**1**
**Name**	**Schemer , Lea**
**Affiliation**	**Rheinland-Pfälzische Technische Universität Kaiserslautern-Landau**
**Date**	**15-Dec-2025**
**COI**	

I have reviewed both the response letter and the revised paper. The authors have carefully addressed my comments and thoroughly revised the manuscript. I am pleased with the updates and thank the authors for their hard work on the revision.

**Reviewer**	**2**
**Name**	**Camerone, Eleonora Maria**
**Affiliation**	**University of Oxford**
**Date**	**02-Jan-2026**
**COI**	

The authors have adequately addressed the reviewers' concerns, resulting in a substantially improved manuscript. While the manuscript is close to being ready for publication, a few remaining issues still need to be addressed.

- Results in Abstract. It might be helpful to more clearly separate the results related to the validation aim from those related to the prediction analyses.

- Sample size. The lack of an a priori sample size calculation remains insufficiently addressed. While the use of a consecutive clinical sample is pragmatic, the added post-hoc power analysis is of limited interpretive value (i.e., power calculated from observed effect sizes largely reflects the reported p-values). I suggest reporting an effect-size sensitivity analysis – this would provide a clearer indication of whether the study was adequately powered to detect effects of a given magnitude. This is particularly important, considering that there are 11 predictors, and the number of participants substantially decreases at follow-ups, thereby increasing the risk of overfitting a regression model to a small sample. (Relevant paper which could be useful: *Giner-Sorolla R et al., Power to Detect What? Considerations for Planning and Evaluating Sample Size. Pers Soc*

Psychol Rev. 2024 Aug;28(3):27C-301. doi: 10.1177/10888C83241228328.)

- Strengths and limitations section (lines 10-11): *"The results of the longitudinal design and their temporal sequence are in line with a potential causal relationship between expectations and clinical outcomes."* This statement seems too strong. Although the longitudinal design establishes temporal ordering, this alone does not justify causal interpretation. This is particularly true given that the longitudinal analyses are exploratory and possibly underpowered relative to the number of predictors and the available sample size (n = 108), which further limits the strength of any causal claims. A more appropriate interpretation is that these findings are hypothesis-generating and provide a starting point for future studies specifically designed to test the causal relationship between expectations and clinical outcomes.

- The transparency of the manuscript would be enhanced by adding a brief statement in the limitations section of the discussion, clarifying the exploratory nature of the predictive analyses. In particular, the authors should note that the small sample size limits hypothesis-driven inference and that these analyses are intended to be exploratory and hypothesis-generating, with no prespecified hypotheses.

- I remain unconvinced that the association between improvement expectations and desire for pain relief provides meaningful evidence for construct validity. Although the authors argue that the two constructs are theoretically related, they also describe them as conceptually distinct (cognitive appraisal versus affective motivation). Importantly, theory does not clearly predict the direction of their association: patients may report a high desire for pain relief alongside either high or low expectations of improvement (e.g., high desire for pain relief but low expectations of improvement following previous treatment failure). Because these constructs could plausibly be positively, negatively, or not at all associated, the observed correlation is not informative for construct validity. As currently presented, this analysis does not strengthen the validity argument and may instead introduce conceptual ambiguity. Unless the authors can provide a clear theoretical rationale or empirical evidence supporting an expected direction of association, I would recommend removing this analysis and the related comments.

- Line 31: *"Moreover, in **the regression analyses**, the GEEE's current improvement item yielded similar results to the pain intensity and pain-related disability outcomes, further confirming the validity of the instrument."* Shouldn't this refer to the correlations, rather than the regression analyses? (i.e., how current improvement is correlated to the other variables).

- I was not able to find the Figures file.

**Reviewer**	**3**
**Name**	**Purtill, Helen**
**Affiliation**	**University of Limerick, Mathematics and Statistics**
**Date**	**11-Dec-2025**
**COI**	

The authors have addressed some of my previous concerns and their revisions have improved the manuscript and the transparency of the statistical analyses and results. However, I still have concerns on the study design and analyses as outlined below:

1. In the limitations section on page 15, lines 20 - 22, it is stated that this study was not designed as a validation study "However, as this investigation was based on a naturalistic clinical sample rather than a study designed exclusively for validation purposes, the evidence for validity must be interpreted within this context." This should be added to the Abstract under the aim. Currently the Abstract on page 2 lines 10/11 states "The present study aims to test the validity and applicability of this new instrument in patients with chronic... " but it can't truly test validity because it was not designed to do so. It should be a statement more in line with what was given in the limitations section - that the study is an exploration of GEEE in a clinical setting which may provide evidence for the usefulness and validity of GEEE based on a naturalistic clinical sample of people living with chronic pain.

2. A supplementary table showing baseline differences between those who completed the 3 timepoints and those who did not and were lost to follow-up should be provided. On pg 9, lines 1-3, It is acknowledged that those lost to follow-up at T3 were more likely to have higher pain, disability and anxiety at baseline (that is those with worse pain were more likely to leave) - this needs to be acknowledged when discussing the regression results for T3 in the results section.

2. The updated Table 1 is a very good addition to the paper with median (IQR) given for those measures found to be skewed. There are very strong floor effects evident for GEEE worsening and GEEE side effects which were mentioned in the limitations section but need to be acknowledged/discussed in reporting on the descriptive statistics presented in Table 1 on page 8 and should inform subsequent analyses. For example if at least 75% do not expect worsening (upper quartile = 0 on the scale) and at least 75% of participants express 9 or higher on the desire for pain relief scale there is very little variation on these scales. Variation across a scale is desirable for correlational analysis - otherwise, as in GEEE worsening, what we are seeing is a very high percentage of participants are answering that scale in the same way - making it impossible to measuring any improvements on that scale.

3. The floor effects of GEEE worsening and GEEE side effects and ceiling effects of desire for pain should be taken into account when discussing any results from correlations or

regressions. On page 9 under "Validity GEEE expectations" a small negative correlation is seen as evidence of discriminant validity - but this could also be a result of the floor/ceiling effects/almost zero variability. Also in the discussion on pg 13, line 24 - 27 "The GEEE's improvement expectation item was consistently associated with desire for pain relief, providing supportive evidence of construct validity, acknowledging that the two constructs are theoretically related but not identical." - How can this provide evidence for construct validity when over 75% of the sample score 9 or 10 on the desire for pain scale?

4. On page 10 GEEE subjective improvement outcome correlated with pain and pain-related disability - but would this not be expected given that pain and pain-related disability are self-reported measures from participants? What does GEEE subjective improvement offer in addition to the information from pain and pain-related disability measurements?

5. Pg 10 "Treatment Expectations over Time" is followed by "Correlations: GEEE Previous Experience and Baseline Expectations" which are assessed at baseline and are not over time.

6. Pg 10 line 34/35 "Linear Mixed Model Analyses. Linear mixed model analyses yielded the same pattern of results, confirming the findings from the complete case repeated-measures ANOVAs " - supporting rather than confirming as Linear mixed models do assume that missing values are missing at random (MAR) which may not strictly hold - and the T3 drop-out analysis suggests it is not MAR.

7. On pages 12 and 13 Prediction of Pain and Pain-Related Disability at T3 was reported and it was found that baseline GEEE improvement expectations were predictive of pain and pain-related disability. But given that at T3 those who dropped out were significantly more likely to have higher baseline pain, anxiety, depression and desire for pain relief - it would be important to comment/discuss how this might impact the results at T3.

8. The limitations in the Abstract needs to include reference to loss to follow-up at T2 and T3 and also the ceiling/floor effects observed in some of the GEEE scales and desire for pain.

VERSION 2 - AUTHOR RESPONSE

Reviewer: 1
Dr. Lea Schemer , Rheinland-Pfälzische Technische Universität Kaiserslautern-Landau
Comments to the Author:
I have reviewed both the response letter and the revised paper. The authors have carefully addressed my comments and thoroughly revised the manuscript. I am pleased with the updates and thank the authors for their hard work on the revision.

Response: We thank the reviewer for the positive feedback and for the careful evaluation of the revised manuscript.

Reviewer: 2
Dr. Eleonora Maria Camerone, University of Oxford
Comments to the Author:
See pdf attached

The authors have adequately addressed the reviewers' concerns, resulting in a substantially improved manuscript. While the manuscript is close to being ready for publication, a few remaining issues still need to be addressed.

1. Results in Abstract. It might be helpful to more clearly separate the results related to the validation aim from those related to the prediction analyses.

Response: We thank the reviewer for this helpful suggestion. We have revised the Results section of the abstract to more clearly distinguish between findings related to the validation aim and those related to the prediction analyses by explicitly signposting these two sets of results.

In-text changes: Abstract, Results (p. 2, lines 26 and 30/31): Regarding validation, at baseline, improvement expectations correlated weakly with expectations of worsening and side effects (Rho≈−.14 to −.15, p = .028–.034). Negative previous treatment experiences were associated with current expectations of worsening and side effects (Rho≈.35–.41, $p \leq .002$). The generic GEEE items on current treatment effects correlated with changes in clinical outcomes (pain intensity, pain-related disability) (|r| ≈ .17–.56, all $p \leq .043$). Regarding prediction, treatment expectations remained stable, while pain intensity (η^2G=.076, $p < .001$) and pain-related disability (η^2G=.037, $p < .001$) decreased over time. Higher improvement expectations and side-effect expectations at baseline predicted subjective improvement at 3 weeks (R^2=.18; f^2 =.21, p=.014), while both higher improvement expectations and higher expectations of worsening predicted subjective improvement at 16 weeks (R^2=.29, f^2=.42, p=.010). Baseline improvement expectations also predicted lower pain intensity and less pain-related disability at 16 weeks (R^2≈.50–.59; f^2≈.98–1.44, all $p \leq .010$).

2. Sample size. The lack of an a priori sample size calculation remains insufficiently addressed. While the use of a consecutive clinical sample is pragmatic, the added post-hoc power analysis is of limited interpretive value (i.e., power calculated from observed effect sizes largely reflects the reported p-values). I suggest reporting an effect-size sensitivity analysis – this would provide a clearer indication of whether the study was adequately powered to detect effects of a given magnitude. This is particularly important, considering that there are 11 predictors, and the number of participants substantially decreases at follow- ups, thereby increasing the risk of overfitting a regression model to a small sample. (Relevant paper which could be useful: *Giner-Sorolla R et al., Power to Detect What? Considerations for Planning and Evaluating Sample Size. Pers Soc Psychol Rev. 2024 Aug;28(3):276-301. doi: 10.1177/10888683241228328.)*

Response: We are grateful to the reviewer for this comment, as well as for pointing us to the recent methodological paper, which was very helpful in guiding the revision in line with current best practice. In response, we removed the post-hoc power analyses and conducted effect-size sensitivity analyses for the regression models at both follow ups. The sensitivity analyses indicate that the models were sufficiently powered to detect effects of moderate magnitude in both follow ups, while smaller effects may not have been detectable. We have added these changes in the Methods section, present the results in the Results section and briefly incorporated their implications into the strengths and limitations section of the Discussion to clarify the scope and interpretation of the findings.

In-text changes:
Methods (p.8, lines 16-17): Effect-size sensitivity analyses were conducted for the regression models at both follow ups (36).
Results (p.11, lines 10-13): Effect-size sensitivity analyses (α=.05, 80% power, 11 predictors) indicated sensitivity to moderate effects at the first follow-up (N=135; minimum detectable Cohen's f^2=0.14) and continued sensitivity to moderate effects at the second follow-up despite attrition (N = 102; f^2=0.18).
Discussion (p. 16, lines 24-25): Sensitivity analyses indicated adequate power to detect moderate effects at both follow-ups, while smaller effects may have gone undetected.

3. Strengths and limitations section (lines 10-11): *"The results of the longitudinal design and their temporal sequence are in line with a potential causal relationship between expectations and clinical outcomes."* This statement seems too strong. Although the longitudinal design establishes temporal ordering, this alone does not justify causal interpretation. This is particularly true given that the longitudinal analyses are exploratory and possibly underpowered relative to the number of predictors and the available sample size (n = 108), which further limits the strength of any causal claims. A more appropriate interpretation is that these findings are hypothesis-generating and provide a starting point for future studies specifically designed to test the causal relationship between expectations and clinical outcomes.

Response: We agree with the reviewer that temporal ordering alone does not justify causal interpretation. In line with this comment and in line with the editor's comment on the same point, we have removed this point from the "Strengths and limitations" section of the abstract. Instead, and in response to comments from Reviewer 3, we now include a methodological limitation in this section referring to substantial loss to follow-up at T2 and T3 and floor and ceiling effects in some measures.

In-text changes: Abstract Strengths and Limitations point 4 (p. 3, lines 13-15): Substantial loss to follow-up and limited variance in some variables (GEEE expectation of worsening, GEEE expectation of side effects, desire for pain relief) may have limited statistical power and generalizability.

4. The transparency of the manuscript would be enhanced by adding a brief statement in the limitations section of the discussion, clarifying the exploratory nature of the predictive analyses. In particular, the authors should note that the small sample size limits hypothesis-driven inference and that these analyses are intended to be exploratory and hypothesis-generating, with no prespecified hypotheses.

Response: We have revised the limitations section of the Discussion to explicitly clarify the exploratory and hypothesis-generating nature of the predictive analyses. Specifically, we now note that the relatively small sample size limits hypothesis-driven inference and that no prespecified hypotheses were tested. This addition improves transparency regarding the interpretation of the regression analyses.

In-text changes: Discussion (p.16, lines 25-27): Given the relatively small sample size, these predictive analyses should be interpreted as exploratory and hypothesis-generating rather than hypothesis-driven, as no prespecified hypotheses were tested.

5. I remain unconvinced that the association between improvement expectations and desire for pain relief provides meaningful evidence for construct validity. Although the authors argue that the two constructs are theoretically related, they also describe them as conceptually distinct (cognitive appraisal versus affective motivation). Importantly, theory does not clearly predict the direction of their association: patients may report a high desire for pain relief alongside either high or low expectations of improvement (e.g., high desire for pain relief but low expectations of improvement following previous treatment failure). Because these constructs could plausibly be positively, negatively, or not at all associated, the observed correlation is not informative for construct validity. As currently presented, this analysis does not strengthen the validity argument and may instead introduce conceptual ambiguity. Unless the authors can provide a clear theoretical rationale or empirical evidence supporting an expected direction of association, I would recommend removing this analysis and the related comments.

Response: We thank the reviewer for this careful and constructive critique. At the outset of the study, we hypothesized that improvement expectations and desire for pain relief would be positively associated, based on the assumption that both constructs reflect patients' engagement with treatment and perceived need for change. However, upon further reflection, we agree that the theoretical relationship between these constructs does not imply a clearly specified or unidirectional association, as plausible scenarios exist for positive, negative, or absent correlations. In addition, the distribution

of desire for pain relief in our sample was highly skewed, with limited variance, which further constrains the interpretability of correlational analyses. We have therefore removed the correlations with desire for pain relief from the construct validity argument and deleted the related text in this section to improve conceptual clarity and methodological rigor. The correlations between improvement expectations and desire for pain relief are now only reported under "Correlations: GEEE Expectations, Prior Experience and Related Variables" on page 10, lines 20-22 and are no longer presented as a validity indicator.

In text changes: Results (p.10, lines 20-22): The GEEE improvement expectation item correlated significantly with desire for pain relief at baseline (Rho(217)=.345, p<.001), at T2 (Rho(138)=.331, p<.001), and at T3 (Rho(106)=.309, p=.001).

6. Line 31: *"Moreover, in* ***the regression analyses****, the GEEE's current improvement item yielded similar results to the pain intensity and pain-related disability outcomes, further confirming the validity of the instrument."* Shouldn't this refer to the correlations, rather than the regression analyses? (i.e., how current improvement is correlated to the other variables).

Response: We thank the reviewer for this helpful observation and agree that different aspects of validity are supported by different analytic approaches. We have therefore revised this section to more clearly distinguish between correlational and regression-based evidence. Specifically, we now attribute support for convergent validity to the observed associations between subjective improvement and changes in pain intensity and pain-related disability. In addition, we clarify that the regression analyses indicate that the GEEE current improvement item is embedded within a similar pattern of predictors—particularly improvement expectations—as the other clinical outcome measures. This consistency across models is now framed as evidence for criterion-related (predictive) validity rather than as an extension of the correlational findings.

In-text changes: Discussion (p. 13, lines 33-36): Significant correlations between GEEE treatment effect items and changes in clinical outcomes confirm the convergent validity of the items; greater perceived improvement was associated with reduced pain intensity and pain-related disability, while subjective worsening corresponded with poorer clinical outcomes. In addition, regression analyses showed that the GEEE current improvement item shared a similar pattern of significant predictors—most notably improvement expectations—with pain intensity and pain-related disability outcomes, supporting the criterion-related (predictive) validity of the instrument (see below).

7. I was not able to find the Figures file.

Response: Thank you for pointing this out. The Figures file has been uploaded again with the revised submission; no changes were made to the figures.

Reviewer: 3
Dr. Helen Purtill, University of Limerick
Comments to the Author:
The authors have addressed some of my previous concerns and their revisions have improved the manuscript and the transparency of the statistical analyses and results. However, I still have concerns on the study design and analyses as outlined below:

1. In the limitations section on page 15, lines 20 - 22, it is stated that this study was not designed as a validation study "However, as this investigation was based on a naturalistic clinical sample rather than a study designed exclusively for validation purposes, the evidence for validity must be interpreted within this context." This should be added to the Abstract under the aim. Currently the Abstract on page 2 lines 10/11 states "The present study aims to test the validity and applicability of this new instrument in patients with chronic... " but it can't truly test validity because it was not designed to do so. It should be a statement more in line with what was given in the limitations section - that the study

is an exploration of GEEE in a clinical setting which may provide evidence for the usefulness and validity of GEEE based on a naturalistic clinical sample of people living with chronic pain.

Response: We thank the reviewer for this helpful suggestion. We have revised the aim in the Abstract to reflect that the study explores the GEEE in a clinical setting and may provide preliminary evidence for its usefulness and validity, in line with the limitations described in the manuscript.

In-text changes:
Abstract (p.2, lines 10-12): "The present study aims to explore the GEEE in a naturalistic clinical sample of people seeking treatment for chronic pain, which may provide preliminary evidence for its validity and applicability."

2. A supplementary table showing baseline differences between those who completed the 3 timepoints and those who did not and were lost to follow-up should be provided. On pg 9, lines 1-3, It is acknowledged that those lost to follow-up at T3 were more likely to have higher pain, disability and anxiety at baseline (that is those with worse pain were more likely to leave) - this needs to be acknowledged when discussing the regression results for T3 in the results section.

Response: We have now added supplementary tables comparing baseline characteristics of participants who completed the follow-up assessments and those lost to follow-up at T2 and T3 (Appendix A). In addition, we have included a statement in the Results section to acknowledge potential attrition bias when presenting the T3 regression analyses, and we have expanded the Discussion to explicitly address the implications of selective dropout for the interpretation of the longitudinal findings.

In-text changes:
Appendix A (Additional statistical information) now provides the comparisons between completers and non-completers at T2 and T3. The tables are referred to in text in the results section (p.8, line 37).

Results (p.11, lines 8-10): "For the T3 analyses, it should be noted that participants lost to follow-up had higher baseline pain, depression, anxiety, and desire for pain relief, indicating potential attrition bias in these models."

Discussion, Strengths and Limitations (p. 16, lines 17-21): "Finally, attrition was substantial, which is common in naturalistic longitudinal data collection; at T3, non-completers had higher baseline symptom severity in terms of pain intensity, depression and anxiety and desire for pain relief, indicating that this was a more seriously affected subgroup. This pattern suggests potential attrition bias, such that the T3 analyses may overrepresent patients with better baseline functioning. The results may therefore overestimate the strength of associations between predictors and outcomes that are more pronounced in patients with better baseline functioning and may not generalize to more severely affected patients. Moreover, because the regression analyses at T3 were based on a reduced sample with multiple predictors, the risk of model overfitting cannot be ruled out. Our findings on the association between expectations and treatment outcomes are correlational rather than causal in nature, as we did not manipulate expectations directly. To draw causal conclusions about this association, experimental studies are required."

3. The updated Table 1 is a very good addition to the paper with median (IQR) given for those measures found to be skewed. There are very strong floor effects evident for GEEE worsening and GEEE side effects which were mentioned in the limitations section but need to be acknowledged/discussed in reporting on the descriptive statistics presented in Table 1 on page 8 and should inform subsequent analyses. For example if at least 75% do not expect worsening (upper quartile = 0 on the scale) and at least 75% of participants express 9 or higher on the desire for pain relief scale there is very little variation on these scales. Variation across a scale is desirable for correlational analysis - otherwise, as in GEEE worsening, what we are seeing is a very high

percentage of participants are answering that scale in the same way - making it impossible to measuring any improvements on that scale.

Response: We thank the reviewer for this important methodological point. We have now explicitly acknowledged the strong floor effects for the GEEE expectation of worsening and side effects scales, as well as the ceiling effect for desire for pain relief, in the Results section when presenting the descriptive statistics in Table 1. In addition, we have included a brief caution in the Results section prior to the regression analyses, noting restricted variance in these measures. Finally, we have expanded the Discussion to address the implications of restricted variance for correlational and regression analyses, highlighting that limited variability may distort associations and limits the robustness of findings involving these scales.

In-text changes:
Results (p. 8, lines 31-33): “As shown in Table 1, strong floor effects were observed for the GEEE worsening and GEEE side effects scales, while a pronounced ceiling effect was evident for desire for pain relief, indicating restricted variance for these measures.”
Results (p 11, lines 6-8): “Restricted variance in some scales (GEEE expectation of worsening, GEEE expectation of side effects, desire for pain relief) should be considered when interpreting these analyses.”
Discussion (p.15, lines 32-35): “In this sample, low expectations of worsening and side effects suggest strong floor effects, while desire for pain relief showed a ceiling effect, indicating restricted variance that may distort associations in correlational and regression analyses and limit the robustness of findings involving these scales.”
Discussion (p. 15, lines 14-17): Given the model’s modest explanatory power, floor effects for expectations of worsening, and selective attrition at T3, the findings should be interpreted with caution.

4. The floor effects of GEEE worsening and GEEE side effects and ceiling effects of desire for pain should be taken into account when discussing any results from correlations or regressions. On page 9 under "Validity GEEE expectations" a small negative correlation is seen as evidence of discriminant validity - but this could also be a result of the floor/ceiling effects/almost zero variability. Also in the discussion on pg 13, line 24 - 27 "The GEEE’s improvement expectation item was consistently associated with desire for pain relief, providing supportive evidence of construct validity, acknowledging that the two constructs are theoretically related but not identical." - How can this provide evidence for construct validity when over 75% of the sample score 9 or 10 on the desire for pain scale?

Response:
We have revised the interpretation of the correlations reported under “Validity GEEE expectations” to explicitly acknowledge that strong floor and ceiling effects limit the interpretability of these associations, and we describe the internal validity evidence as preliminary in the Discussion. In addition, we have expanded the Discussion to note that the observed floor effects may have implications for future research, including the need to explore alternative measurement approaches or subgroup analyses to better capture variability in expectations of worsening and side effects. Furthermore, we have removed the desire for pain relief – improvement expectations correlation as an indicator of construct validity in the whole manuscript. This decision was informed both by the pronounced ceiling effect of this measure in the present sample and by another reviewer’s concern regarding the conceptual distinction between desire for pain relief and treatment expectations.

In-text changes:
Results (p.9, lines 11- 15): At baseline, improvement expectations showed small negative correlations with expectations of worsening (Rho(217)=-0.15, p=.028) and side effect expectations (Rho(217)=-.14, p=.034), which may tentatively support discriminant validity, although the restricted variance observed for these scales may have attenuated or artificially influenced correlation estimates.

Additionally, expectations of worsening and of side effects were positively associated with one another (Rho(217)=.31, p<.001), which may support convergent validity under the same constraints.

Discussion (p.15, lines 35-37): Future research could explore alternative measurement strategies or subgroup analyses to better capture variability in expectations of worsening and side effects and to identify patient characteristics associated with these expectations.

5. On page 10 GEEE subjective improvement outcome correlated with pain and pain-related disability - but would this not be expected given that pain and pain-related disability are self-reported measures from participants? What does GEEE subjective improvement offer in addition to the information from pain and pain-related disability measurements?

Response: We agree that correlations between subjective improvement and changes in pain-related outcomes are expected, given that all measures are based on patient self-report. This was our reasoning for using these associations as indicators of convergent validity. Importantly, however, the GEEE subjective improvement item is not intended to measure current symptom severity, but rather a retrospective, global appraisal of perceived change due to treatment. It captures perceived treatment effect rather than symptom intensity per se, which is conceptually distinct from symptom measurement. As such, it captures a patient-centred evaluation that integrates multiple aspects of the treatment experience, including symptom change, coping, side effects, and personal expectations. Although the present study was conducted in a chronic pain sample, the GEEE was designed as a generic assessment tool that can be applied across different clinical conditions and treatment contexts, including settings where no established or comparable outcome measures are available. This generic nature is intended to facilitate future research on treatment expectations and outcomes across diverse interventions and to enhance comparability between studies.

6. Pg 10 "Treatment Expectations over Time" is followed by "Correlations: GEEE Prior Experience and Baseline Expectations" which are assessed at baseline and are not over time.

Response: We thank the reviewer for this comment. We agree that the original section heading was too narrow for the range of analyses presented. We have therefore revised the section heading to **"GEEE Treatment Expectations: Antecedents, Trajectories and Prediction of Outcomes"** in both the Results (p.10, line 15) and Discussion (p.14, line 16) sections to more accurately reflect the content of the analyses.

7. Pg 10 line 34/35 "Linear Mixed Model Analyses. Linear mixed model analyses yielded the same pattern of results, confirming the findings from the complete case repeated-measures ANOVAs " - supporting rather than confirming as Linear mixed models do assume that missing values are missing at random (MAR) which may not strictly hold - and the T3 drop-out analysis suggests it is not MAR.

Response: Indeed, this is an important methodological clarification. We have revised the wording in the main text to replace "confirming" with "supporting" and have added a sentence in Appendix E acknowledging the MAR assumption underlying linear mixed-effects models and its potential limitation given the observed pattern of attrition at T3.

In-text changes:
Results (p.10, line 34): **Linear Mixed Model Analyses.** Linear mixed model analyses yielded the same pattern of results, supporting the findings from the complete case repeated-measures ANOVAs (see: Appendix E).

Appendix E: To address reviewer feedback and to account for missing data resulting from participant dropout, linear mixed-effects models (LMMs) were conducted for each dependent variable across the three measurement time points. LMMs allow for the inclusion of all available observations without requiring complete data from all participants, thereby providing a more robust estimation of longitudinal changes. Results for each variable are presented below. These results should be

interpreted in light of the assumption that missing data are missing at random (MAR), which may be violated given that dropout was associated with baseline symptom severity.

8. On pages 12 and 13 Prediction of Pain and Pain-Related Disability at T3 was reported and it was found that baseline GEEE improvement expectations were predictive of pain and pain-related disability. But given that at T3 those who dropped out were significantly more likely to have higher baseline pain, anxiety, depression and desire for pain relief - it would be important to comment/discuss how this might impact the results at T3.

Response: We agree with the reviewer that selective dropout at T3 is an important limitation for the interpretation of the prediction analyses. We have therefore addressed this point at several levels in the manuscript. First, in the Results section when presenting the regression analyses, we added a cautionary statement noting both restricted variance in some scales and potential attrition bias, explicitly referring to the higher baseline symptom severity among participants lost to follow-up (as also reported in response to Comment 2). Second, in the Discussion of the regression findings, we expanded the interpretation to consider how selective attrition may have biased the models toward healthier patients and potentially inflated observed associations. Finally, we expanded on this issue in the general strengths and limitations section, where we discuss the broader implications of selective attrition for the robustness and generalizability of the T3 findings.
In addition, following Reviewer 2's suggestion (Comment 2), we conducted effect-size sensitivity analyses for the longitudinal regression models. These analyses indicated sensitivity to effects of moderate magnitude despite the reduced sample size at T3. The results of the sensitivity analyses are now reported in the Results section and their implications are briefly discussed in the manuscript to further qualify the interpretation of the T3 findings.

In-text changes:
Results (p.11, lines 8-13): ***Regressions: Baseline Expectations Predict Clinical Outcomes.***
Only significant models are reported. For all regression results, see Appendix C. Restricted variance in some scales (GEEE expectation of worsening, GEEE expectation of side effects, desire for pain relief) should be considered when interpreting these analyses. For the T3 analyses, it should be noted that participants lost to follow-up had higher baseline pain, depression, anxiety, and desire for pain relief, indicating potential attrition bias in these models. Effect-size sensitivity analyses (α=.05, 80% power, 11 predictors) indicated sensitivity to moderate effects at the first follow-up (N=135; minimum detectable Cohen's f^2=0.14) and continued sensitivity to moderate effects at the second follow-up despite attrition (N = 102; f^2=0.18).

Discussion (p. 15, lines 14-17): Given the model's modest explanatory power, floor effects for expectations of worsening, and selective attrition at T3, the findings should be interpreted with caution.

Discussion, Strengths and Limitations (p. 16, lines 17-21 and lines 23-26): This pattern suggests potential attrition bias, such that the T3 analyses may overrepresent patients with better baseline functioning. The results may therefore overestimate the strength of associations between predictors and outcomes that are more pronounced in patients with better baseline functioning and may not generalize to more severely affected patients. Moreover, because the regression analyses at T3 were based on a reduced sample with multiple predictors, the risk of model overfitting cannot be ruled out. Sensitivity analyses indicated adequate power to detect moderate effects at both follow-ups, while smaller effects may have gone undetected. Given the relatively small sample size, these predictive analyses should be interpreted as exploratory and hypothesis-generating rather than hypothesis-driven, as no prespecified hypotheses were tested.

9. The limitations in the Abstract needs to include reference to loss to follow-up at T2 and T3 and also the ceiling/floor effects observed in some of the GEEE scales and desire for pain.

Response: We have revised the "Strengths and limitations of this study" section in the abstract to explicitly include substantial loss to follow-up and limited variance in some measures (GEEE expectation of worsening, GEEE expectation of side effects, desire for pain relief), and highlight their potential implications for interpretation.

In-text changes:
Abstract, Strengths and limitations (p.3, lines 13-15): Substantial loss to follow-up and limited variance in some variables (GEEE expectation of worsening, GEEE expectation of side effects, desire for pain relief) may have limited statistical power and generalizability.

VERSION 3 - REVIEW

**Reviewer**	**2**
**Name**	**Camerone, Eleonora Maria**
**Affiliation**	**University of Oxford**
**Date**	**23-Mar-2026**
**COI**	

The authors have responded thoughtfully to my comments and have made comprehensive revisions to the manuscript. I am satisfied with the changes and appreciate the effort they have put into this revision.

**Reviewer**	**3**
**Name**	**Purtill, Helen**
**Affiliation**	**University of Limerick, Mathematics and Statistics**
**Date**	**16-Mar-2026**
**COI**	

The authors have careful considered and addressed my previous concerns in their revisions to the manuscript.
